# Single-crystalline hole-transporting layers for efficient and stable organic light-emitting devices

**DOI:** 10.1038/s41377-024-01484-4

**Published:** 2024-06-07

**Authors:** Gao-Da Ye, Ran Ding, Su-Heng Li, Lei Ni, Shu-Ting Dai, Nian-Ke Chen, Yue-Feng Liu, Runda Guo, Lei Wang, Xian-Bin Li, Bin Xu, Jing Feng

**Affiliations:** 1 State Key Laboratory of Integrated Optoelectronics, College of Electronic Science and Engineering, Jilin University, 2699 Qianjin Street, 130012 Changchun, China; 2 State Key Laboratory of Supermolecular Structures and Materials, The Institute of Theoretical Chemistry, Jilin University, 2699 Qianjin Street, 130012 Changchun, China; 3 Wuhan National Laboratory for Optoelectronics, Huazhong University of Science and Technology, 430074 Wuhan, China

**Keywords:** Organic LEDs, Optoelectronic devices and components

## Abstract

Efficient charge-carrier injection and transport in organic light-emitting devices (OLEDs) are essential to simultaneously achieving their high efficiency and long-term stability. However, the charge-transporting layers (CTLs) deposited by various vapor or solution processes are usually in amorphous forms, and their low charge-carrier mobilities, defect-induced high trap densities and inhomogeneous thickness with rough surface morphologies have been obstacles towards high-performance devices. Here, organic single-crystalline (SC) films were employed as the hole-transporting layers (HTLs) instead of the conventional amorphous films to fabricate highly efficient and stable OLEDs. The high-mobility and ultrasmooth morphology of the SC-HTLs facilitate superior interfacial characteristics of both HTL/electrode and HTL/emissive layer interfaces, resulting in a high Haacke’s figure of merit (FoM) of the ultrathin top electrode and low series-resistance joule-heat loss ratio of the SC-OLEDs. Moreover, the thick and compact SC-HTL can function as a barrier layer against moisture and oxygen permeation. As a result, the SC-OLEDs show much improved efficiency and stability compared to the OLEDs based on amorphous or polycrystalline HTLs, suggesting a new strategy to developing advanced OLEDs with high efficiency and high stability.

## Abstract (web summary)

Organic single-crystalline films are employed as the hole-transporting layers for developing advanced OLEDs with high efficiency and high stability.

## Graphical abstract

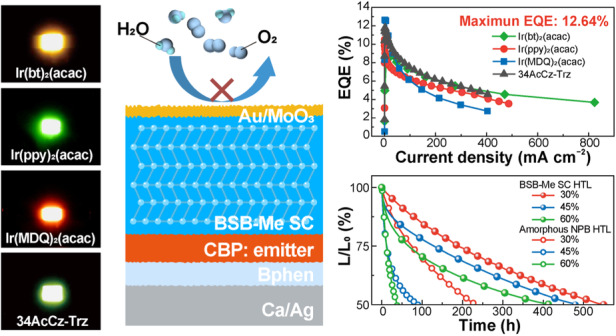

## Introduction

Organic light-emitting device (OLED) technology has made incredible progress in the past few decades and stepped into commercialization for flat-panel displays and solid-state lighting sources^1–9^. Among the various functional layers in the OLED structure, charge-transporting layers (CTLs) as the key component for charge-carrier injection/transport, play a vital role in determining the device performance^10–13^. The CTLs deposited by conventional vapor or solution process are usually in amorphous forms with random orientation of the molecules, which result in their inherently low charge-carrier mobilities in the range of 10^−3^–10^−6^ cm^2^ V^−1^ s^−1^ and thereafter a strict thickness of only tens of nanometers in the OLED structure. Such a strict thickness of the amorphous CTLs cannot cover the substrate uniformly, and their inhomogeneous thickness would induce the formation of shunting paths between the electrodes and consequent degradation of the device performance^14,15^. Meanwhile, the electrode/organic interface adhesion is an important factor for the device stability, and it has been demonstrated that the dark-spot growth and joule heating can be suppressed by increasing the interfacial adhesion to avoid electrode delamination^16–19^. Unfortunately, amorphous thin films usually possess a rough surface that will induce a non-homogenous metallic electrode deposition with poor electrode/organic adhesion, leading to pinholes for moisture and oxygen penetration as well as electrode delamination. Employing thicker CTLs is a possible solution to overcome the above issues by covering the substrate more uniformly and improving the surface morphologies. However, the thick CTLs inevitably give rise to high operating voltages because of their low mobilities and generate Joule heating, accelerating device degradation during operation. Two-micrometer-thick perovskite films have been employed as the CTLs in OLEDs, and the low driving voltage was maintained by the high mobility of the perovskites^14^. Nevertheless, the rough surface, specific fabrication process, and inherent instability of the polycrystalline perovskite films limited their applications toward commercialized OLEDs. One-micrometer-thick amorphous organic thin film also has been used as the CTL, while a specific Ohmic contact formation by constructing a multilayered structure between the emissive layer and electrode as well as a relatively high mobility of the amorphous CTL is needed, which means rather limited choice of the materials^15^. Therefore, thicker CTLs with high mobilities, low thickness variations, and smooth surface morphologies are highly required for both high efficiency and long-term stability of the OLEDs and are still an ongoing challenge.

Organic single-crystalline semiconductors (OSCs) have long-range ordered molecular arrangement and low impurity content, leading to their superior charge-transport properties^20–28^. The highest charge-carrier mobility approaching ~43 cm^2^ V^−1^ s^−1^ has been reported from a rubrene single-crystal-based transistor, which is several orders of magnitude higher than that of amorphous thin films^29^. Different from the amorphous or polycrystalline films with rough, incompact, and uneven morphologies, the OSCs possess outstanding properties from the point of view of their morphologies, such as atomic or molecular-level ultrasmooth surfaces with uniform thickness. Moreover, a more compact nature with dense packing of molecules in the OSCs is favorable for the long-term stability of the OLEDs by impeding moisture and oxygen permeation. So far, the applications of the OSCs in OLEDs (SC-OLEDs) as functional layers have been mostly focused on their light-emitting properties^24–27^. For example, Ding et al. have demonstrated three-primary-color and white OLEDs by using molecule-doped OSCs as the emissive layers^30,31^. Highly polarized OLEDs were also realized by using the OSC emitters due to their highly aligned molecular orientation and inherent anisotropic properties^32,33^. These unique features of the OSCs propose their great potential as the CTLs to provide an alternative method to realize thicker OLEDs with high efficiency and high stability, however, it has not been fully explored yet.

In this work, a 400-nm-thick *p*-type OSC with high-quality single-crystallinity, ultrasmooth surface morphology, and superior charge-transport property is utilized as the hole-transporting layer (HTL) in the SC-OLED. The hole mobility of organic SC films is around 0.2 cm^2^ V^−1^ s^−1^ along the out-of-plane direction corresponding to the charge-carrier transport in the vertical OLED structure, which is much higher than those of amorphous and polycrystalline films (~10^−3^–10^−4^ cm^2^ V^−1^ s^−1^). Both the top and bottom interfacial characteristics of the HTLs are improved due to the ultrasmooth surface morphology and ordered molecular orientation of the SC films, leading to a high FoM of the top ultrathin electrode and stronger interaction between the HTL and emissive layer. A low series-resistance joule-heat loss ratio of only 13.14% is obtained, benefiting from the superior mobility and improved interfacial characteristics of the SC films. Furthermore, dense molecular packing of the thick SC-HTL can function as an internal moisture and oxygen barrier layer to protect other organic functional layers, which enables to effectively improve the operational stability. The SC-HTLs are feasible to the OLEDs based on various emitters, and an external quantum efficiency (EQE) of 12.64% is obtained for the SC-OLEDs with a phosphorescent emitter, which is the highest value for SC-OLEDs reported so far.

## Results

### Characteristics of BSB-Me SC-HTL for OLEDs

**Fig. 1:**
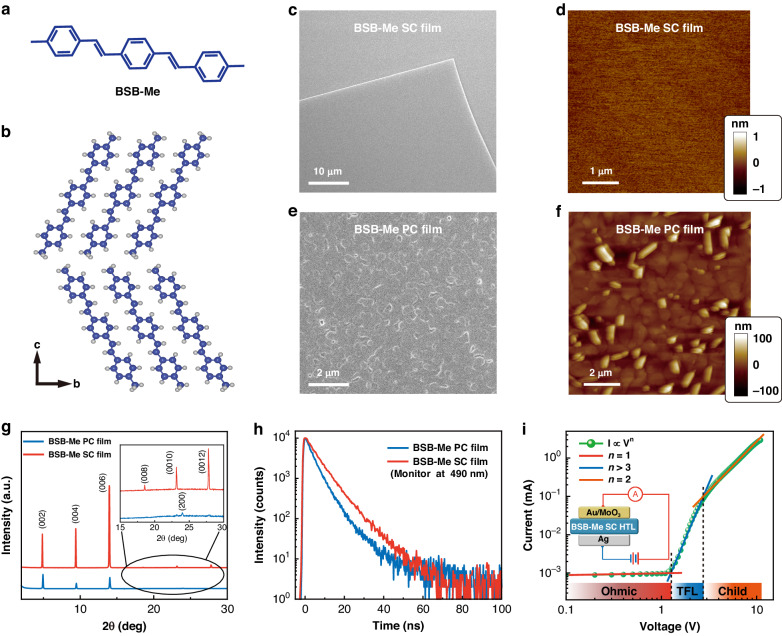
Characterizations of BSB-Me SC and PC films. **a** Molecular formula of BSB-Me and **b** molecular packing diagram of BSB-Me crystal. SEM and AFM images of BSB-Me SC (**c**, **d**) and PC (**e**, **f**) films. **g** XRD patterns of BSB-Me SC and PC films. The inset shows more detailed XRD patterns in the range of 15°–30°. **h** PL decay curves of BSB-Me SC and PC films. **i** Current–voltage curve of the SC hole-only device showing three different dependence regions (*I*–*V*^*n*^): a linear ohmic region (*I*−*V*, *n* = 1, red line) at low bias, a trap-filled region starting at *V*_TFL_ (*I*–*V*^*n*^, *n* > 3, blue line), and a trap-free Child’s region at a high bias (*I*−*V*^2^, *n* = 2, orange line). The inset shows the schematic architecture of the SC hole-only device

Herein, we choose a typical *p*-type 1,4-bis(4-methylstyryl) benzene (BSB-Me) crystal as the HTL (Fig. 1a), in which the molecules are stacked layer by layer in a dense herringbone packing (Fig. 1b), resulting in strong overlap between the π-orbitals of adjacent molecules and thereafter high charge-carrier mobility and high stability^34,35^. Slice-like BSB-Me SC films were grown by the physical vapor transport method. Meanwhile, polycrystalline (PC) films of the BSB-Me can be obtained by thermal deposition at a slow rate via strong π–π stacking and characterized for comparison^36^. The CTL surface morphology is an important factor in facilitating charge-carrier injection and transport^10,11^. Firstly, the surface morphologies of BSB-Me SC and PC films were investigated by scanning electron microscopy (SEM) and atomic force microscopy (AFM) images, which are shown in Fig. 1c–f. From Fig. 1d, the AFM probe has scanned across the BSB-Me SC surface with an area of 5 μm × 5 μm, exhibiting an ultrasmooth surface with a root-mean-squared (RMS) roughness value of only 0.108 nm. On the contrary, there appeared obvious grains for the PC film with a much larger RMS surface roughness approaching 20.3 nm. X-ray diffraction (XRD) measurements were carried out to gain an insight into the crystalline structure, as shown in Fig. 1g. For BSB-Me SC film, a series of multi-order and sharp diffraction peaks in the XRD diagram can be indexed to the (00*h*) lattice planes, implying an SC nature with pure crystallographic orientation^34,35^. Single-crystal X-ray crystallographic results revealed that BSB-Me belongs to the space group of *Pbca* with crystal parameters of *a* = 7.362(5) Å, *b* = 5.883(4) Å and *c* = 38.95(2) Å (CCDC 708328). BSB-Me molecules adopt a standing growth model in which their molecular long axes are inclined to the crystal *ab*-plane with an oblique angle of almost 60°. However, different XRD diffraction peaks corresponding to other crystal planes can be observed from BSB-Me PC film (inset of Fig. 1g), as well as lower intensity and broader width of the peaks, implying its polycrystallinity. As shown in Fig. S1, the full width at half maximum (FWHM) of (002) plane of the BSB-Me PC film was found to be 0.098°, which was broader than that of the BSB-Me SC film (0.062°). The recombination dynamics of photoexcited species in BSB-Me SC and PC films were investigated by time-resolved photoluminescence (TRPL) spectroscopy. The PL decay curves were presented in Fig. 1h and monitored at the emission wavelength of 490 nm. Their corresponding PL spectra were measured and shown in Fig. S2. The decay times of BSB-Me SC and PC films can be estimated to be 6.89 and 4.76 ns, respectively. The longer PL lifetime of BSB-Me SC film further implies high single-crystallinity and suppressed trap states inside. Top-view fluorescence photographs show that BSB-Me SC film exhibits brighter fluorescence emission from both surface and edge due to its high quality of single-crystallinity as compared to the PC film (Fig. S3).

To evaluate the carrier transport behavior, the hole mobility of BSB-Me SC film was determined by the space-charge-limited current (SCLC) method based on the hole-only device with a configuration of Au (12 nm)/MoO_3_ (5 nm)/BSB-Me SC film/Ag (80 nm) (inset of Fig. 1i)^37–39^. The mobilities of BSB-Me PC film as well as the commonly used HTL of amorphous N,N’-Bis(naphthalen-1-yl)-N,N’-bis(phenyl)-benzidine (NPB) film, were also investigated for comparison (Fig. S4 and Supplementary Note 1). The current–voltage characteristic of a log–log plot of the BSB-Me SC hole-only device showed three distinct regions (Fig. 1i). The trap density (*n*_t_) can be estimated from the trap-filled limit voltage (*V*_TFL_) using the following equation^40^:

1 $${n}_{{\rm {t}}}=\frac{2{V}_{{{ {TFL}}}}{\varepsilon }_{0}{\varepsilon }_{{\rm {r}}}}{e{L}^{2}}$$

where *L* is the thickness of the active layer, *e* is the electron charge, and *ε*_0_ and *ε*_r_ correspond to the vacuum permittivity and the relative dielectric constant of organic semiconductors with a value of ~3, respectively. A lower trap density of 1.28 × 10^15^ cm^−3^ was obtained for BSB-Me SC film in comparison to those of BSB-Me PC and NPB films (4.48 × 10^16^ and 5.48 × 10^16^ cm^−3^, respectively) due to its perfect single-crystallinity. When the bias voltage was above 2.5 V, the current–voltage characteristic of the BSB-Me SC film operated in the Child’s region (Fig. 1i, orange line), which can be well fitted by the Mott–Gurney law^38–41^

2 $$J=\frac{9}{8}{\varepsilon }_{0}{\varepsilon }_{{\rm {r}}}\mu \frac{{V}^{2}}{{L}^{3}}$$

where *J* is the current density, *μ* is the charge-carrier mobility, and *V* is the applied voltage. A high hole mobility of 0.18 cm^2^ V^−1^ s^−1^ for BSB-Me SC film can be derived from the curve fitting, as shown in Fig. S4a. The Child region was not reached for both BSB-Me PC and NPB films (Fig. S4b), due to the device failure that probably caused by the joule heat at high current density. Therefore, the time-of-flight (TOF) measurements were further used to investigate the hole mobilities of BSB-Me SC, BSB-Me PC, and NPB films^40^, and they were estimated to be 0.21, 2.73 × 10^−3^, and 3.31 × 10^−4^ cm^2^ V^−1^ s^−1^, respectively (Fig. S5, Table S1 and Supplementary Note 2). Combined with SCLC measurements, the hole mobility of BSB-Me SC film was two to three orders of magnitude higher than those of BSB-Me PC and amorphous NPB films, respectively.

### EL performances of the SC-OLEDs

**Fig. 2:**
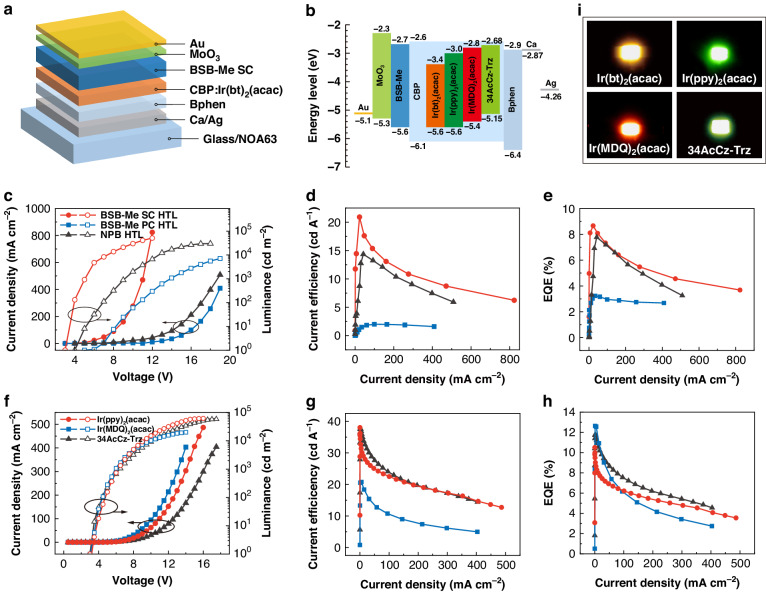
EL performances of the SC-OLEDs. Schematic illustration of device structure (**a**) and the corresponding energy-level diagram (**b**) of the SC-OLED. Plots of current density–voltage–luminance (**c**), current efficiency–current density (**d**), and EQE–current density (**e**) of OLEDs based on SC, PC, and amorphous HTLs with Ir(bt)_2_(acac) emitter. Plots of current density–voltage–luminance (**f**), current efficiency–current density (**g**), and EQE-current density (**h**) of SC-OLEDs based on Ir(ppy)_2_(acac), Ir(MDQ)_2_(acac), and 34AcCz-Trz emitters. **i** The photographs of the operating SC-OLEDs based on four different emitters

In view of the high-quality single-crystallinity and superior charge-transport properties of BSB-Me SC films, OLEDs with BSB-Me SC films as the HTLs were fabricated. A facile template-stripping method was utilized in the fabrication of SC-OLEDs, where electrodes and other functional layers can be directly deposited on both the top and bottom sides of the crystal slices, enabling compact contact and efficient charge-carrier injection^41^. The details of the fabrication process of SC-OLEDs were described in the “Materials and methods” section and shown in Fig. S6. The device structure is Ag (80 nm)/Ca (3 nm)/4,7-Diphenyl-1,10-phenanthroline (Bphen; 40 nm)/4,4’-Bis(carbazol-9-yl)biphenyl: Bis(2phenylbenzothiazolato) (acetylacetonate)iridium(III) (CBP: Ir(bt)_2_(acac); 20 nm)/ BSB-Me SC film/MoO_3_ (5 nm)/Au (12 nm), as shown in Fig. 2a. Here, CBP: Ir(bt)_2_(acac) was used as the emissive layer, in which Ir(bt)_2_(acac) was a typical phosphorescence-based emitter working as the dopant. And, CBP functioned as a host material doped with Ir(bt)_2_(acac) at a doping concentration of 5 wt%^42^. Other functional layers of Bphen and MoO_3_ were also adopted for the electron transport layer (ETL) and hole injection layer (HIL), respectively. The highest occupied molecular orbital (HOMO) and lowest unoccupied molecular orbital (LUMO) of BSB-Me were reported to be 5.6 and 2.7 eV, respectively, which have taken into consideration the energy-level matching^34,43^. As inferred from the corresponding energy-level diagram in Fig. 2b, hole injection from MoO_3_ to CBP will be prevented by a large energy barrier of 0.8 eV between MoO_3_ and CBP. The insertion of the BSB-Me with the HOMO level of 5.6 eV between the MoO_3_ and CBP will promote the hole injection and transport from MoO_3_ to CBP. Being the HTL of OLEDs, BSB-Me SC thickness was controlled at around 400 nm. The corresponding AFM image and height profile are provided in Fig. S7. In order to verify the superiority of SC-HTLs, OLEDs with HTLs of amorphous NPB and BSB-Me PC films were fabricated for comparison. The thicknesses of the amorphous NPB and BSB-Me PC films were optimized to be 40 nm. The device structures of OLEDs with three different HTLs are shown in Fig. S8. Current density–voltage–luminance, current efficiency–current density, and external quantum efficiency (EQE)–current density curves of OLEDs based on these three different HTLs are shown in Fig. 2c–e. Relative increase in the current density of BSB-Me SC HTL-based OLEDs can be attributed to the high mobility and ultrasmooth morphology of the BSB-Me SC-HTLs. Also, rough BSB-Me PC films with submicron-size grains may bring defects and traps to impede charge-carrier transport and injection, which makes the current density–voltage characteristics of BSB-Me PC HTL-based OLEDs worse than that of NPB devices. The maximum luminance, current efficiency, and EQE of BSB-Me SC HTL-based OLEDs can reach up to 51310 cd m^−2^, 20.92 cd A^−1^, and 8.66%, which were much superior to those of amorphous NPB and BSB-Me PC HTL-based OLEDs. The EL performances of OLEDs based on the SC, PC, and amorphous HTLs are also summarized in Table 1 for comparison. Even at the brightness of 1000 cd m^−2^, the device performances of BSB-Me SC HTL-based OLEDs remained at a relatively high value of 8.11% for EQE, 14.46 cd A^−1^ for current efficiency, and a lower driving voltage of 5.1 V. In particular, the higher charge-carrier mobility of BSB-Me SC film enables the HTL with a thickness of ~400 nm, which is one order of magnitude thicker than those of amorphous NPB and BSB-Me PC HTLs, and even affords a lower operating voltage of the OLEDs without reducing EL performances. Moreover, to further examine the EL performances, SC-OLED with different BSB-Me SC-HTL thicknesses were fabricated, in which the thinner SC HTL of ~200 nm was used for comparison. As shown in Fig. S9, the current efficiency and EQE of the 200-nm-thick BSB-Me SC HTL-based OLED is lower than that of the device based on a 400-nm-thick BSB-Me SC HTL. The higher hole mobility of BSB-Me SC HTL will lead to more accumulation of excess holes at the interface of the HTL/emissive layer or within the emissive layer for the thinner SC HTL, which might account for the lower EL performances of the SC-OLEDs with the 200-nm-thick SC HTL. The above results suggest that using BSB-Me SC film as the HTL is a potential route to realize ultrathick OLEDs with promoted EL performances.

**Table 1:** The EL performances of OLEDs based on three different HTLs

Device	*V*_on_ (V)	Maximum			@1000 cd m^−2^		
		Luminance (cd m^−2^)	CE (cd A^−1^)	EQE (%)	*V*_1000_ (V)	CE (cd A^−1^)	EQE (%)
BSB-Me SC HTL	3.0	51310	20.92	8.66	5.1	14.46	8.11
BSB-Me PC HTL	5.0	7138	2.01	3.23	14.0	1.48	3.19
NPB HTL	4.0	30160	14.35	7.78	9.2	6.44	3.57

*V*_on_ is the turn-on voltage of OLEDs at a luminance of 1 cd m^−2^. *V*_1000_ is the driving voltage at 1000 cd m^−2^. CE is the current efficiency

To evaluate the feasibility of SC-HTLs in the OLEDs, various emitters including another two phosphorescent emitters of Bis[2-(2-pyridinyl-N)phenyl-C](acetylacetonato)iridium(III) (Ir(ppy)_2_(acac)) and Bis(2-methyldibenzo[f,h]quinoxaline) (acetylacetonate)iridium(III) (Ir(MDQ)_2_(acac)), and a thermally activated delayed fluorescence (TADF) emitter of 5-(4-(4,6-diphenyl-1,3,5-triazin-2-yl)phenyl)-13,13-dimethyl-8-phenyl-8,13-dihydro-5H-indolo[3,2-a]acridine (34AcCz-Trz) were employed in the SC-OLEDs to construct a similar device structure with the decreased top electrode thickness from 12 to 8 nm. The doping concentrations of these three emitters in the CBP host were optimized to be 5 wt%^44^. The emissive-layer thicknesses of Ir(ppy)_2_(acac), Ir(MDQ)_2_(acac), and 34AcCz-Trz were optimized to be 40, 40, and 30 nm, respectively. Current density–voltage–luminance, current efficiency–current density, and EQE–current density curves are shown in Fig. 2f–h. The maximum EQEs of the SC-OLEDs based on Ir(ppy)_2_(acac), Ir(MDQ)_2_(acac), and 34AcCz-Trz were 10.50%, 12.64%, and 11.80%, respectively. The apparent efficiency roll-off of the SC-OLEDs can be ascribed to the long excited state lifetime of the phosphorescent and TADF emitters. The long excited state lifetime may induce triplet–triplet quenching, triplet–polaron quenching, and singlet–triplet quenching. The exciton quenching will be significant at high current density, which leads to the efficiency roll-off. The other reason is high-mobility BSB-Me SC HTL (~0.2 cm^2^ V^−1^ s^−1^), which is several orders of magnitude higher than that of amorphous Bphen films (~3 × 10^−4^ cm^2^ V^−1^ s^−1^)^45^. Therefore, the unbalanced hole and electron transport characteristic may give rise to hole accumulation within the emissive layer, inducing efficiency decrease at high current density. Figure 2i presents the photographs of the operating SC-OLEDs based on the four different emitters, which achieve bright and homogeneous surface EL emissions of yellow, green, red, and yellow-green light. Figure S10 shows the EL spectra with the emission peaks of 518, 540, 564, and 600 nm, corresponding to the Ir(ppy)_2_(acac), 34AcCz-Trz, Ir(bt)_2_(acac), and Ir(MDQ)_2_(acac) emitters, respectively. The EL performances of our SC-OLEDs have been compared with previous light-emitting devices based on organic single-crystalline or polycrystalline materials, which are summarized in Table S2. To the best of our knowledge, the EQE of 12.64% is the highest value for SC-OLEDs reported so far, confirming the technical feasibility of using organic single-crystalline films as HTL materials for high-performance SC-OLEDs.

### Interfacial characteristics of both SC-HTL/top electrode and SC-HTL/bottom emissive layer

**Fig. 3:**
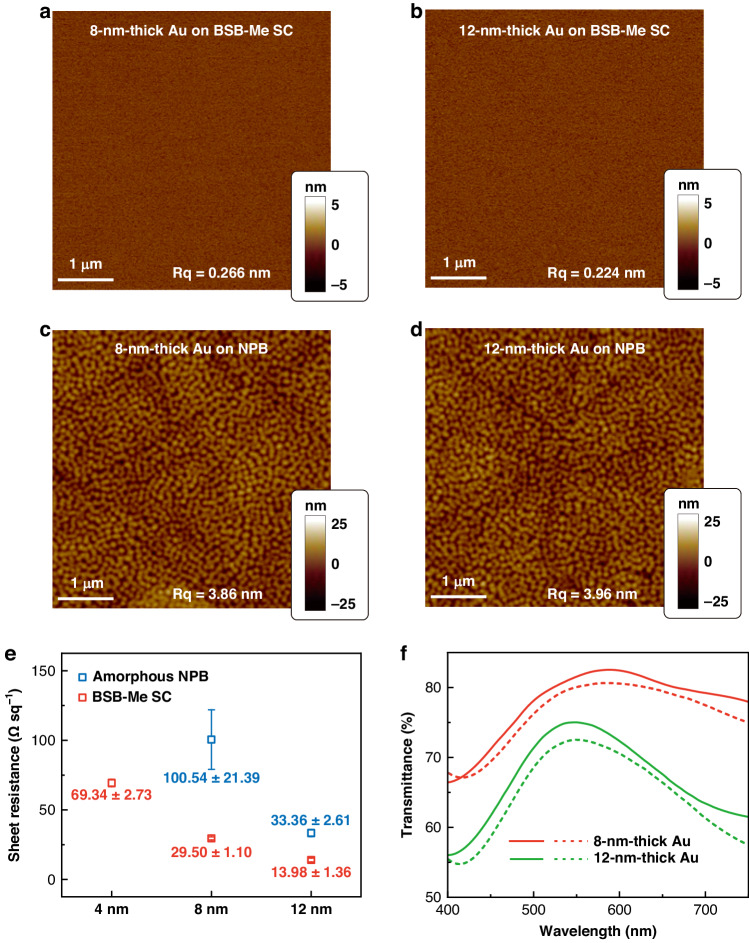
Characteristics of top electrode deposited on SC-HTL. AFM images of 8-nm-thick (**a**) and 12-nm-thick (**b**) Au anodes on BSB-Me SC films and 8-nm-thick (**c**) and 12-nm-thick (**d**) Au anodes on amorphous NPB films. **e** Comparison of sheet resistance (*R*_sh_) of Au anodes on BSB-Me SC and amorphous NPB films with different thicknesses. **f** Transmittance of Au anodes on BSB-Me SC (solid lines) and amorphous NPB (dashed lines) films with 8 and 12 nm thickness

The interfacial characteristics within the device structure are important to determine the EL performances of the OLEDs. It is noteworthy that BSB-Me SC films with ultrasmooth surface morphology provide a good platform to realize ultrathin overlying functional layers. The surface morphologies of BSB-Me SC and amorphous NPB films have been investigated by the AFM images with RMS roughness values of 0.108 and 3.13 nm, respectively, as shown in Figs. 1d and S11. Then, the top Au anode, together with a 5-nm-thick MoO_3_ HIL, was directly thermal-deposited onto the BSB-Me SC surface corresponding to the SC-OLEDs. As can be seen from the AFM images in Fig. 3a, b, the ultrathin Au anodes with 8- and 12-nm thickness deposited on BSB-Me SC films exhibit comparable surface roughness (RMS of 0.2–0.3 nm) to the crystal surface. On the contrary, 8- and 12-nm-thick Au deposited on amorphous NPB films presented a rough surface with an RMS roughness up to 3.86–3.96 nm (Fig. 3c, d). Figure 3e summarizes the sheet resistance (*R*_sh_) of ultrathin Au anodes deposited on the surface of BSB-Me SC or amorphous NPB films. The Au on the amorphous NPB exhibited much higher *R*_sh_ than those on the BSB-Me SC, and the 4-nm Au on amorphous NPB seems insulated. The Au on BSB-Me SC showed much better conductivity, and their *R*_sh_ increased from 13.98 to 29.50 and 69.34 Ω sq^−1^ with a decreasing Au thickness from 12 to 8 and 4 nm. The *R*_sh_ of Au anodes on BSB-Me PC films was also investigated. The 12-nm-thick Au on BSB-Me PC showed a much higher value of about 80.38 Ω sq^−1^ and the 8-nm-thick Au on BSB-Me PC is insulated due to the rough BSB-Me PC films with submicron-size grains. The 8- and 12-nm-thick Au on BSB-Me SC showed a comparable conductivity to the commercial indium tin oxide (ITO) film (~36 Ω sq^−1^). Their transmittance (*T*) at the wavelengths of 400–750 nm was further measured (Fig. 3f), and the 8- and 12-nm-thick Au on the BSB-Me SC achieved relatively higher transmission of 82% and 74%, respectively, at 564 nm than those on amorphous NPB (80% and 72%). The transmittance at the wavelength of 564 nm corresponds to the EL emission peak of Ir(bt)_2_(acac) emitter. Then, the FoM was determined by FoM = *T*^10^/*R*_sh_ at 564 nm^46,47^. They were estimated to be 4.66 × 10^−3^ and 3.52 × 10^−3^ Ω^−1^ for the 8- and 12-nm-thick Au on BSB-Me SC, respectively, whereas they were 1.07 × 10^−3^ and 1.12 × 10^−3^ Ω^−1^ on the amorphous NPB. The Haacke’s FoM as a function of transmittance wavelength corresponding to EL emission peaks of Ir(ppy)_2_(acac), 34AcCz-Trz, Ir(bt)_2_(acac), and Ir(MDQ)_2_(acac) emitters have been summarized in Table S3. The much higher FoM, as well as ultrasmooth morphologies of the Au anodes on the BSB-Me SC films, can be attributed to the ultrasmooth surface morphologies of the OSCs and would be beneficial to efficient charge-carrier injection from the anode to the HTL. The EL performances of the SC-OLEDs based on ultrathin Au anodes with different thicknesses were compared and shown in Fig. S12. As expected, the SC-OLEDs with 8-nm-thick Au showed a superior EL performance. It should be noted that the NPB HTL-based OLEDs with 8-nm-thick Au anode eventually failed due to the rough surface of the amorphous NPB and the induced discontinuity of the Au anode (Fig. S13). Nevertheless, the SC-OLEDs can work normally even when the thickness of the Au anode is decreased to 4 nm. The improved continuity and ultrasmooth morphology of the ultrathin Au anode on the SC-HTL would facilitate not only the superior optoelectrical properties of the top electrode but also the interfacial adhesion between the top electrode and the SC-HTL.

**Fig. 4:**
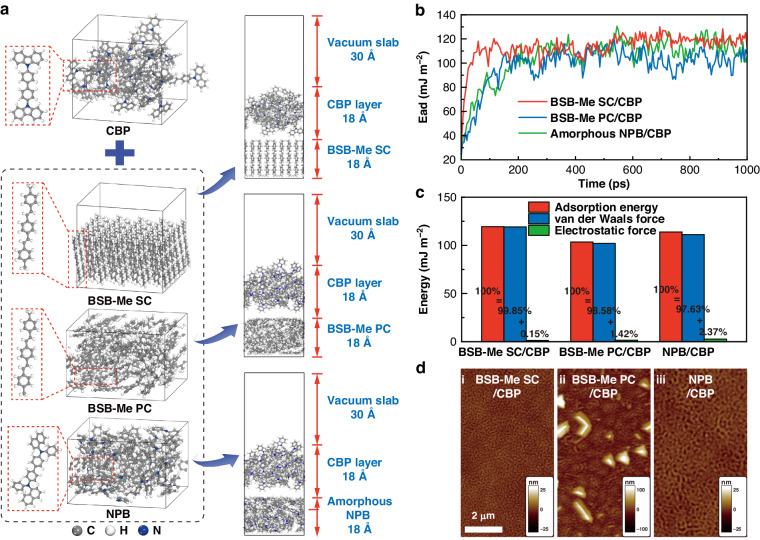
Interfacial characteristics of SC-HTL/bottom emissive layer. **a** The established model of interaction systems of the CBP layer together with BSB-Me SC, BSB-Me PC, and amorphous NPB surfaces, respectively. **b** Calculated adsorption energy of BSB-Me SC/CBP, BSB-Me PC/CBP, and amorphous NPB/CBP interaction systems by MD simulation. **c** Histogram of adsorption energy with the contributions from van der Waals force and electrostatic force. **d** AFM images of thermal-deposited CBP layer atop BSB-Me SC, BSB-Me PC, and amorphous NPB surfaces, respectively

Molecular dynamics (MD) simulation was employed to investigate the interfacial property of the SC-HTL/bottom emissive layer from a microscopic point of view^48–53^. The molecules of host CBP were taken into account for the simulation because of their high concentration in the emissive layer. Figure 4a illustrates the established model of the interaction system of the BSB-Me SC/CBP interface, and the models of the BSB-Me PC/CBP and amorphous NPB/CBP interfaces were used for comparison. The fluctuation range of temperature and the kinetic, potential, non-bond, and total energy curves were within 5–10% (Fig. S14), implying that the systems have reached their equilibrium state. The atomic configurations of the system at different moments are shown in Figs. S15–17. The interaction strength for the interfaces of BSB-Me SC/CBP (BSB-Me PC/CBP and amorphous NPB/CBP) can be characterized by the adsorption energy (*E*_ad_) and calculated as follows^49,53^:

3 $${E}_{{{\rm {ad}}}}=\frac{\left({E}_{{{\rm {surface}}}}+{E}_{{{\rm {CBP}}}}\right)-{E}_{{{\rm {total}}}}}{A}$$

where *E*_total_ is the total energy of BSB-Me SC/CBP (BSB-Me PC/CBP and amorphous NPB/CBP) interaction system in an equilibrium state, *E*_surface_, and *E*_CBP_ are the energies of BSB-Me SC (BSB-Me PC and amorphous NPB) surface and CBP layer, respectively, and *A* is the sectional area of the interface. The averaged adsorption energy can be estimated to be ~119.37 mJ m^−2^ for BSB-Me SC/CBP, as shown in Fig. 4b, c, including the contributions from van der Waals (vdW) force (119.20 mJ m^−2^) and electrostatic force (0.17 mJ m^−2^). Meanwhile, they are about 103.49 mJ m^−2^ for BSB-Me PC/CBP, including the contributions of 102.02 mJ m^−2^ from vdW force and 1.47 mJ m^−2^ from electrostatic force, and about 113.89 mJ m^−2^ for amorphous NPB/CBP including the contributions of 111.19 mJ m^−2^ from vdW force and 2.70 mJ m^−2^ from electrostatic force. The higher contributions from the vdW force mean that the interaction system is mainly provided by non-bonding interaction^49,53^. AFM measurements were taken to determine the surface morphologies of the thermal-deposited CBP layer on BSB-Me SC, BSB-Me PC, and amorphous NPB (Fig. 4d). The CBP film deposited on the BSB-Me SC surface shows more uniform and continuous morphology than those on BSB-Me PC and amorphous NPB. The greater value of the adsorption energy for the BSB-Me SC/CBP implies the stronger adhesion between the BSB-Me SC surface and CBP layer, which may be originated from the ordered molecular orientation and ultrasmooth surface morphology of the BSB-Me SC film. The improved interfacial characteristics of both the top and bottom interfaces of the SC-HTLs would contribute to sufficient charge-carrier injection and transport within device layers, ensuring better EL performances of SC-OLEDs. Moreover, the improved interfacial adhesion of SC-HTL has an effect on suppressing the delamination of the functional layers within the device, especially the electrode delamination, which would greatly improve the operational stability of the OLEDs. The improved charge-carrier injection and transport at the SC-HTL interfaces, together with the superior charge-transport behaviors of the SC-HTL enable to lower the energy loss from Joule heating^54,55^. The instantaneous slope, i.e., areal differential conductance and resistance, and several electric parameters, such as input power and Joule heating of series-resistance, can be obtained from the current density-voltage-luminance characteristics (Fig. S18) and summarized in Table S4. The calculation of the ratio of series-resistance Joule-heat loss to input power has been described with details in Supplementary Note 3. The BSB-Me SC HTL-based OLEDs possessed a ratio of series-resistance joule-heat loss to input power (13.14%) at the luminance of 1000 cd m^−2^, which was much lower than that of amorphous NPB HTL-based OLEDs (22.67%).

### Operational stability of the SC-OLEDs

**Fig. 5:**
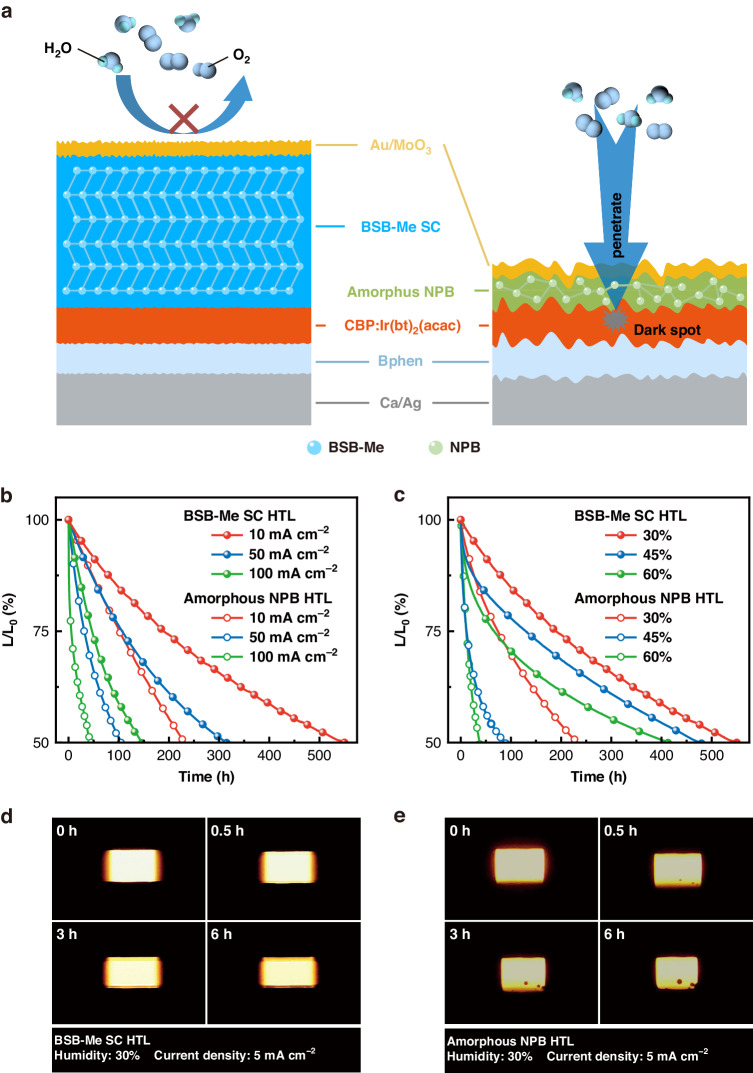
Operational stability of the SC-OLEDs. **a** Schematic illustration of the device structure of the SC-OLED and amorphous OLED. Normalized luminance of SC-OLEDs and amorphous OLEDs as a function of operating time under different driving current densities (**b**) and humidity (**c**). The photographs of the operating SC-OLED (**d**) and amorphous OLED (**e**) at a humidity of 30% and current density of 5 mA cm^−2^ as a function of operating time (unencapsulated and tested in the air)

The enhanced interfacial adhesion, as well as the decreased Joule heating, will be conducive to the operational stability of the SC-OLEDs. The operational stability of the BSB-Me SC and amorphous NPB HTL-based OLEDs was examined in an accelerated condition under different driving current densities and humidity^17^. The duration decaying from initial luminance (100 cd m^−2^) to 50% was defined as the half-lifetime of OLED. Figure 5b, c show the normalized luminance of the OLEDs under different humidity and current density. The SC-OLEDs exhibited higher operating stability than the amorphous OLEDs at both high driving current density and high humidity. The SC-OLEDs exhibited almost three times longer half-lifetimes (550, 315, and 146 h) than those of amorphous OLEDs (226, 105, and 45 h) at the increasing driving current density of 10, 50, and 100 mA cm^−2^, under a constant humidity of 30% (Fig. 5b). The enhanced stability of the SC-OLEDs at high driving current density can be attributed to their lower series-resistance joule-heat loss ratio. The half-lifetimes of the SC-OLEDs gradually decreased from 550 to 479 and 413 h with increasing humidity from 30% to 45% and 60%, respectively, and at a constant driving current of 10 mA cm^−2^. The half-lifetimes of the amorphous OLEDs greatly decreased from 226 to 89 and 37 h, as shown in Fig. 5c. The photographs of operating devices as a function of operating time under the humidity of 30% and current density of 5 mA cm^−2^ are shown in Fig. 5d, e. The dark spots can be observed clearly in the emitting area of the amorphous OLEDs soon after 0.5 h operating, whereas no dark spots were observed for SC-OLEDs even after 6 h operating. It has been demonstrated that the dark-spot formation at the cathode/organic interface of the OLEDs can be ascribed to the cathode delamination by moisture and oxygen penetration through cathode pinholes, which results in water electrolysis-induced bubble formation or crystallized cluster formation of the organic films^16,18^. The ultrasmooth SC surface enables a uniform deposition of the top electrode and compact contact with each other, which is particularly beneficial to increase the electrode/HTL interfacial adhesion, giving rise to the suppressed dark-spot growth in SC-OLEDs. In addition, morphological stability is another key factor that is associated with OLED operational stability. We, therefore, investigated the morphological evolution of BSB-Me SC and amorphous NPB films via the AFM images. As shown in Fig. S19, BSB-Me SC film exhibited superior morphological stability up to 120 h in the atmospheric environment (25 °C and 30% humidity), whereas the amorphous NPB film showed an evident deterioration of morphologies. These results demonstrated that the ultrathick and compact SC-HTLs with dense packing of molecules and high morphological stability may build a barrier layer to prevent the penetration of moisture and oxygen and avoid moisture and oxygen erosion to the emissive layer (Fig. 5a). Therefore, SC-HTL plays a key role in improving the long-term stability of OLEDs by suppressing joule heating, improving the interfacial characteristics within the device, and acting as a barrier layer for moisture and oxygen permeation.

## Discussion

In summary, we developed a strategy to effectively improve the efficiency and stability of OLEDs by employing OSC films with high-quality single-crystallinity and high mobility as the HTLs in OLEDs. The improved interfacial characteristics of the SC-HTL in the OLEDs enabled high FoM and great uniformity of ultrathin top Au anodes, as well as stronger interaction between HTL and bottom emissive layer, leading to efficient charge-carrier injection and transport within the device, and thereafter higher efficiency of the SC-OLEDs. Benefiting from both excellent interfacial characteristics and superior charge-transport properties of the SC-HTL, the low series-resistance joule-heat loss ratio of 13.14% was obtained. Furthermore, dense molecular packing of the thick SC-HTL can function as a moisture and oxygen permeation barrier in the OLEDs, providing a facile pathway to effectively improve operational stability. As a result, the SC-OLEDs with a 400-nm-thick SC-HTL showed higher efficiency and operating stability than those of OLEDs using 40-nm-thick BSB-Me PC or amorphous NPB HTLs. A maximum EQE of 12.64% has been achieved with a phosphorescent emitter, which, to the best of our knowledge, is the highest value reported for SC-OLEDs. This work explored the application of the OSCs as the CTLs in OLEDs and shed light on the future development of high-performance OLEDs.

## Materials and methods

### Growth of BSB-Me SC and PC films

BSB-Me was purchased from Tokyo Chemical Industry Co.; Ltd. The BSB-Me SC films were grown via a physical vapor transport (PVT) method. The quartz boat carrying BSB-Me powder was placed inside a quartz tube and then transferred to a horizontal tube furnace with a dual-temperature zone. The temperatures of the sublimation zone and crystallization zone were set to be 270 and 240 °C, respectively, to achieve a temperature gradient over the quartz tube. Pure argon was used as a protector and carrier for the growth of BSB-Me SC films, and the gas flow was maintained at a rate of 40 mL min^−1^. Buoyancy-driven convection will occur in the horizontal tube as a result of the applied horizontal temperature gradient and dominate the growth of BSB-Me SC films^56^. Three hours later, thin-slice BSB-Me SC films hung on the quartz tube wall at the crystallization zone, which can be transferred to arbitrary substrates for device fabrication. The BSB-Me PC films can be obtained via direct thermal deposition based on precise control of the growth condition at a slow rate of 0.8 Å s^−1^ under the pressure of 5 × 10^−4^ Pa. The BSB-Me PC films were found to contain densely-packed submicron-size grains with a high degree of molecular order, which can be ascribed to the strong π–π stacking during growth^36^.

### Fabrication of the SC-OLEDs

NPB, CBP, and Bphen were purchased from Luminescence Technology Corp and used as received without further purification. The phosphorescence-based emitters, Ir(bt)_2_(acac), Ir(ppy)_2_(acac), and Ir(MDQ)_2_(acac) were also purchased from Luminescence Technology Corp. The TADF-based emitter, 34AcCz-Trz, was supported by the Wuhan National Laboratory for Optoelectronics. The molecular formula of the phosphorescence- and TADF-based emitters were shown in Fig. S20. Octadecyltrichlorosilane (OTS) and NOA63 photoresist were purchased from Sigma-Aldrich and Norland, respectively. In our experiment, the facile template-stripping method was employed to fabricate the SC-OLEDs, as shown in Fig. S6^41^. Firstly, the obtained BSB-Me SC films were transferred onto a pre-prepared hydrophobic SiO_2_/Si substrate by the treatment of OTS. Then, layers of the emissive layer (CBP:Ir(bt)_2_(acac), CBP: Ir(ppy)_2_(acac), CBP: Ir(MDQ)_2_(acac), CBP: 34AcCz-Trz), Bphen, Ca and Ag were thermally deposited onto the crystal surface under a high-vacuum pressure of around 5 × 10^−4^ Pa. The optimal thicknesses of Bphen and emissive layers were determined by comparing the EL performance of a series of SC-OLEDs. After that, a droplet of NOA63 photoresist was dripped on the device and covered by a piece of glass. While the photoresist spread and covered the entire device, it was exposed to a UV lamp and cured after the exposure of 15 minutes. Subsequently, the device can be easily stripped from the SiO_2_/Si substrate and attached to the glass substrate. Relying on this hydrophobic OTS layer, the BSB-Me SC films can be easily peeled off from the SiO_2_/Si substrate and transferred to the glass substrate via NOA63 photoresist. Figure S21 shows the optical images of BSB-Me SC films before and after manual stripping. Apparently, the morphology of the BSB-Me SC film is maintained without destruction and cracks after this mechanical process. And, layers of MoO_3_ and Au were finally deposited onto the other side of BSB-Me SC films. The active area of crystal-based OLEDs was determined by shadow masks with an area of 200 × 300 μm^2^.

### Characterizations and measurements

The top-view photographs of BSB-Me SC and PC films were captured through widefield fluorescence microscopy under UV irradiation. The current density-voltage characteristics of the devices were measured by a Keithley 2400 source meter for the hole-mobility evaluation by the SCLC method. McScience T300 system was used to take the TOF measurements at room temperature. The luminance and EL spectrum of the devices were obtained by a Photo Research PR-788 spectrophotometer. The micro-zone transmittance was achieved by focusing the light of the mercury lamp on the sample surface with a spot size of ~1 mm^2^, calibrating the intensity of the transmitted light connected to an optical fiber spectrometer. The sheet resistance of ultrathin Au electrodes was measured by a four-probe square resistance meter. The thicknesses of BSB-Me SC films and the morphologies of electrodes were characterized using AFM equipment in the tapping mode. The humidity and current density-dependent half-lifetimes of the devices were tested in a chamber by monitoring the decay of device luminance. And, the chamber humidity was controlled via a humidifier.

### Models and simulations

The molecular dynamics (MD) simulations were performed using the condensed-phase optimized molecular potential for atomistic simulation studies (COMPASS II) force field and Facite module of Materials Studio software^48–53^. The model of the interaction system of BSB-Me SC (BSB-Me PC and amorphous NPB) and CBP layer was constructed using the Amorphous Cell module. The BSB-Me crystal belongs to the *Pbca* space group with the crystal parameters of *a* = 7.362(5) Å, *b* = 5.883(4) Å and *c* = 38.95(2) Å from CCDC. One molecular layer of BSB-Me (18 Å) was used as the substrate and the surface cell was created from the (001) lattice plane. And the CBP layer with randomly orientated molecules was inserted 18 Å above the BSB-Me crystal (BSB-Me PC and amorphous NPB with randomly orientated molecules) surface to build the BSB-Me SC/CBP (BSB-Me PC/CBP and amorphous NPB/CBP) interface model. The cubic simulation box of the CBP layer with periodic boundary conditions reached equilibrium after a series of geometry optimization, annealing simulation and dynamics calculation. There was a vacuum slab set with a height of 30 Å to avoid the interaction between BSB-Me SC (BSB-Me PC and amorphous NPB) and CBP across the periodic boundary condition. The simulation was run for 1000 ps under a constant temperature of 558 K according to the thermal deposition process. Then, MD simulations of the interactive layer model were actualized in the NVT ensemble using an Andersen thermostat with a time step of 1 fs. The non-bonding interaction in the BSB-Me SC/CBP (BSB-Me PC/CBP and amorphous NPB/CBP) interface model, including the electrostatic force and vdW force, were computed using the Ewald summation method and an atom-based summation method, respectively, with a cutoff radius of 12.5 Å. The last 100 frames were adopted to calculate the adsorption energy.

### Supplementary information

Supporting information

## Data Availability

All data needed to evaluate the conclusions in the paper are present in the paper and/or the Supplementary Materials.

## References

[1] Huang YG (2020). Mini-LED, Micro-LED and OLED displays: present status and future perspectives. Light Sci. Appl..

[2] Uoyama H (2012). Highly efficient organic light-emitting diodes from delayed fluorescence. Nature.

[3] Im Y (2017). Recent progress in high-efficiency blue-light-emitting materials for organic light-emitting diodes. Adv. Funct. Mater..

[4] Chen HW (2018). Liquid crystal display and organic light-emitting diode display: present status and future perspectives. Light Sci. Appl..

[5] Xu RP, Li YQ, Tang JX (2016). Recent advances in flexible organic light-emitting diodes. J. Mater. Chem. C.

[6] Fan XC (2023). Thermally activated delayed fluorescence materials for nondoped organic light-emitting diodes with nearly 100% exciton harvest. SmartMat.

[7] Ji JP (2023). Digitally programmable organic light-emitting tetrodes. SmartMat.

[8] Gao WC (2024). Recent advances in intrinsically stretchable electronic materials and devices. Responsive Mater..

[9] Yu BY (2022). Stretchable and self-healable spoof plasmonic meta-waveguide for wearable wireless communication system. Light Sci. Appl..

[10] Kulkarni AP (2004). Electron transport materials for organic light-emitting diodes. Chem. Mater..

[11] Shahnawaz S (2019). Hole-transporting materials for organic light-emitting diodes: an overview. J. Mater. Chem. C.

[12] Fukagawa H (2016). Novel hole-transporting materials with high triplet energy for highly efficient and stable organic light-emitting diodes. J. Phys. Chem. C.

[13] Jou JH (2015). Approaches for fabricating high efficiency organic light emitting diodes. J. Mater. Chem. C.

[14] Matsushima T (2019). High performance from extraordinarily thick organic light-emitting diodes. Nature.

[15] Liu GH (2022). Efficient and stable one-micrometre-thick organic light-emitting diodes. Nat. Photonics.

[16] Scholz S (2015). Degradation mechanisms and reactions in organic light-emitting devices. Chem. Rev..

[17] Swayamprabha SS (2021). Approaches for long lifetime organic light emitting diodes. Adv. Sci..

[18] Schaer M (2001). Water vapor and oxygen degradation mechanisms in organic light emitting diodes. Adv. Funct. Mater..

[19] Tyagi P (2016). Degradation of organic light emitting diode: heat related issues and solutions. Synth. Met..

[20] Hotta S (2014). Organic single-crystal light-emitting field-effect transistors. J. Mater. Chem. C.

[21] Ding R (2019). Organic single-crystalline semiconductors for light-emitting applications: recent advances and developments. Laser Photonics Rev..

[22] Zhang CC, Chen PL, Hu WP (2016). Organic light-emitting transistors: materials, device configurations, and operations. Small.

[23] Wang CL (2018). Organic semiconductor crystals. Chem. Soc. Rev..

[24] Liu J (2015). High mobility emissive organic semiconductor. Nat. Commun..

[25] Qin ZS (2023). Organic semiconductor single-crystal light-emitting transistors. Adv. Opt. Mater..

[26] Gao C (2023). Harvesting triplet excitons in high mobility emissive organic semiconductor for efficiency enhancement of light-emitting transistors. Adv. Mater..

[27] Li QB (2023). Dibenzothiophene sulfone-based ambipolar-transporting blue-emissive organic semiconductors towards simple-structured organic light-emitting transistors. Angew. Chem. Int. Ed..

[28] Guan YS (2022). A high mobility air-stable n-type organic small molecule semiconductor with high UV-visible-to-NIR photoresponse. Light Sci. Appl..

[29] Yamagishi M (2007). High-mobility double-gate organic single-crystal transistors with organic crystal gate insulators. Appl. Phys. Lett..

[30] Ding R (2017). Highly efficient three primary color organic single-crystal light-emitting devices with balanced carrier injection and transport. Adv. Funct. Mater..

[31] Ding R (2019). High-color-rendering and high-efficiency white organic light-emitting devices based on double-doped organic single crystals. Adv. Funct. Mater..

[32] An MH (2022). Highly polarized emission from organic single-crystal light-emitting devices with a polarization ratio of 176. Optica.

[33] Ding R, Ye GD, Feng J (2023). Recent advances in linearly polarized emission from organic light-emitting diodes. Appl. Phys. Lett..

[34] Nakanotani H (2008). Blue-light-emitting ambipolar field-effect transistors using an organic single crystal of 1,4-Bis(4-methylstyryl)benzene. Appl. Phys. Express.

[35] Kabe R (2009). Effect of molecular morphology on amplified spontaneous emission of bis-styrylbenzene derivatives. Adv. Mater..

[36] Yasuda T (2006). Organic field-effect transistors based on oligo-p- phenylenevinylene derivatives. Jpn. J. Appl. Phys..

[37] Chu TY, Song OK (2007). Hole mobility of *N*,*N’*-bis(naphthalen-1-yl)-*N*,*N’*-bis(phenyl) benzidine investigated by using space-charge-limited currents. Appl. Phys. Lett..

[38] Dong QF (2015). Electron-hole diffusion lengths >175 μm in solution-grown CH_3_NH_3_PbI_3_ single crystals. Science.

[39] Zhumekenov AA (2016). Formamidinium lead halide perovskite crystals with unprecedented long carrier dynamics and diffusion length. ACS Energy Lett..

[40] Wang H (2011). Cyano-substituted Oligo(*p*-phenylene vinylene) single crystals: a promising laser material. Adv. Funct. Mater..

[41] Ding R (2014). Fabrication and characterization of organic single crystal-based light-emitting devices with improved contact between the metallic electrodes and crystal. Adv. Funct. Mater..

[42] Jang JH (2018). Orange phosphorescent Ir(III) complexes consisting of substituted 2-phenylbenzothiazole for solution-processed organic light-emitting diodes. Org. Electron..

[43] An MH (2020). Well-balanced ambipolar organic single crystals toward highly efficient light-emitting devices. Adv. Funct. Mater..

[44] Zhang Q (2019). Integrating TADF luminogens with AIE characteristics using a novel acridine-carbazole hybrid as donor for high-performance and low efficiency roll-off OLEDs. J. Mater. Chem. C.

[45] Xu W (2008). The estimation of electron mobility of 4,7-diphyenyl-1, 10-phenanthroline using space-charge-limited currents. Solid State Commun..

[46] Zhang TR (2022). Transparent ultrathin Ag nanomesh electrode fabricated by nanosphere lithography for organic light-emitting devices. Appl. Phys. Lett..

[47] Kang MS (2018). Synergetic effects of ligand exchange and reduction process enhancing both electrical and optical properties of Ag nanocrystals for multifunctional transparent electrodes. Nanoscale.

[48] Du H, Miller JD (2007). Adsorption states of amphipathic solutes at the surface of naturally hydrophobic minerals: a molecular dynamics simulation study. Langmuir.

[49] Tong ZF, Xie YB, Zhang YH (2018). Molecular dynamics simulation on the interaction between polymer inhibitors and β-dicalcium silicate surface. J. Mol. Liq..

[50] Yoo D (2019). A molecular dynamics study on the interface morphology of vapor-deposited amorphous organic thin films. Phys. Chem. Chem. Phys..

[51] Kim YH (2020). Molecular-scale strategies to achieve high efficiency and low efficiency roll-off in simplified solution-processed organic light-emitting diodes. Adv. Funct. Mater..

[52] Li ZH (2020). Molecular deposition condition dependent structural and charge transport properties of CBP films. Comput. Mater. Sci..

[53] Zeng JP (2022). Molecular dynamics simulation of the adsorption properties of graphene oxide/graphene composite for alkali metal ions. J. Mol. Graph. Model..

[54] Yang JJ (2022). High-efficiency blue-emission crystalline organic light-emitting diodes sensitized by “hot exciton” fluorescent nanoaggregates. Sci. Adv..

[55] Sun PF (2023). An efficient solid-solution crystalline organic light-emitting diode with deep-blue emission. Nat. Photonics.

[56] Laudise RA (1998). Physical vapor growth of organic semiconductors. J. Cryst. Growth.

