# Supplementary material for: Single-crystalline hole-transporting layers for efficient and stable organic light-emitting devices
Source: Light Sci Appl. 2024 Jun 7;13:136. doi: 10.1038/s41377-024-01484-4 (PMC11161501; doi:10.1038/s41377-024-01484-4)
Supplement: Supplementary file 1 — Supporting information [file 41377_2024_1484_MOESM1_ESM.docx]

**Supplementary Information for “****Single-crystalline hole-transporting layers for efficient and stable organic light-emitting devices”**

Gao-Da Ye^1^, Ran Ding^1^, Su-Heng Li^1^, Lei Ni^1^, Shu-Ting Dai^2^, Nian-Ke Chen^1^, Yue-Feng Liu^1^, Runda Guo^3^, Lei Wang^3^, Xian-Bin Li^1^, Bin Xu^2^, and Jing Feng^1^

^1^State Key Laboratory of Integrated Optoelectronics, College of Electronic Science and Engineering, Jilin University, 2699 Qianjin Street, Changchun, 130012, China.

^2^State Key Laboratory of Supermolecular Structures and Materials, The Institute of Theoretical Chemistry, Jilin University, 2699 Qianjin Street, Changchun, 130012, China.

^3^Wuhan National Laboratory for Optoelectronics, Huazhong University of Science and Technology, Wuhan, 430074, China.

Correspondence and requests for materials should be addressed to R. D. and J. F. (email: [dingran@jlu.edu.cn](mailto:dingran@jlu.edu.cn); [jingfeng@jlu.edu.cn](mailto:jingfeng@jlu.edu.cn)).

**Supplementary Notes**

**Supplementary Note 1. Analysis of hole-only devices:**

The carrier mobility of BSB-Me SC film as the HTL was examined by fabricating the hole-only device with structure of Au (12 nm)/ MoO_3_ (5 nm)/ BSB-Me SC film/ Ag (80 nm). The space-charge-limited current (SCLC) formula is used to estimate the hole mobility as follow^1, 2, 3^

$J=\frac{9}{8}\varepsilon_{0}\varepsilon_{r}\mu\frac{V^{2}}{L^{3}}$ (1)

where *d* is the thickness of the BSB-Me SC film, and $\varepsilon_{0}$ and $\varepsilon_{r}$ are the vacuum permittivity and relative dielectric constant, respectively. The relative dielectric constant of organic materials is reported to be ~3. Influenced by the energetic disorder, the carrier mobility *μ* is dependent on the electric field and can be expressed by a Poole-Frenkel formula

$\mu\left( E \right)=\mu_{0}\exp\left( \beta\sqrt{E} \right)$ (2)

After combining formula (1) and (2), the field dependent SCLC can be calculated as

$J=\frac{9}{8}\varepsilon_{0}\varepsilon_{r}\frac{E^{2}}{d}\mu_{0}\exp\left( \beta\sqrt{E} \right)$ (3)

where *μ*_0_ is the zero-filed mobility and *β* is Poole-Frenkel factor. Figure S4a shows the field dependence of hole mobility of BSB-Me SC film. The fitted curve of BSB-Me SC film is in good agreement with the experimental data and the current in this region follows a field-dependent SCLC behavior. According to the fitting results, the value of zero-field mobility *μ*_0_ and Poole-Frenkel factor *β* can be obtained to be 1.09×10^−2^ cm^2^ V^−1^ s^−1^ and 0.0028, respectively. For the electric field at 0.1 MV cm^−1^, the estimated hole mobility of BSB-Me SC film reaches 0.18 cm^2^ V^−1^ s^−1^.

**Supplementary Note 2. Time-of-flight (TOF) measurements:**

According to the method, the organic films are sandwiched between two electrodes and holes are photogenerated by short laser pulses at the contact, and drawn apart by the applied electric field. The devices used for the TOF measurements were based on the structures of Ag (80 nm)/ BSB-Me SC (490 nm)/ MoO_3_ (5 nm)/ Au (30 nm), Ag (80 nm)/ BSB-Me PC (500 nm)/ MoO_3_ (5 nm)/ ITO, and Ag (80 nm)/ NPB (500 nm)/ MoO_3_ (5 nm)/ ITO, respectively. By measuring the transit time of photogenerated holes, the mobility *μ* can be determined by the following equation,

$\mu=\frac{d^{2}}{V\times t_{tr}}$ (4)

where *d* is the thickness of the organic film, *V* is the applied bias, and *t_tr_* is the transit time. From the above transient photocurrent spectra, the transit times of BSB-Me SC, BSB-Me PC, and NPB films can be determined to be 1.12×10^−7^, 3.05×10^−7^, and 8.38×10^−7^ s, respectively, with an applied bias of 0.1, 3, and 9 V. Then, hole mobilities of BSB-Me SC, BSB-Me PC, and NPB films can be estimated to be 0.21, 2.73×10^−3^, and 3.31×10^−4^ cm^2^ V^−1^ s^−1^, respectively.

**Supplementary Note 3. Calculation on the ratio of series-resistance Joule-heat loss to input power:**

The Shockley equation was used to describe the actual current-voltage curve of OLEDs^12^:

$I-\frac{\left( V-IR_{s} \right)}{R_{p}}=I_{s}{exp}^{\frac{e\left( V-IR_{s} \right)}{nkT}}$ (5)

where *R*_s_ and *R*_p_ are the series resistance and parallel resistance, respectively, *I*_s_ is the reverse saturation current, *n* is the ideality factor of the light-emitting diode, *k* is the Boltzmann constant, and *T* is the thermodynamic temperature. The interface resistance of device including series resistance and parallel resistance will induce Joule heat, while the OLED is under operation. At a large driving voltage, the parallel resistance (*R*_p_) can be negligible. Only the series resistance (*R*_s_) is taken into consideration for the Joule heat generation (*P_J_*) of the OLED, which is calculated as follows:

$P_{J}=I^{2}R_{s}$ (6)

Here, the series resistance (*R*_s_) can be determined by the slope of the current-voltage curve under high driving voltage. The input power is the product of voltage and current, so the equation is as follows:

$P_{in}=V\times I$ (7)

Therefore, the ratio (*R*) of series-resistance Joule-heat loss to input power of the OLEDs can be calculated from the following equation:

$R=\frac{P_{J}}{P_{in}}$ (8)

**Supplementary Figures**

**
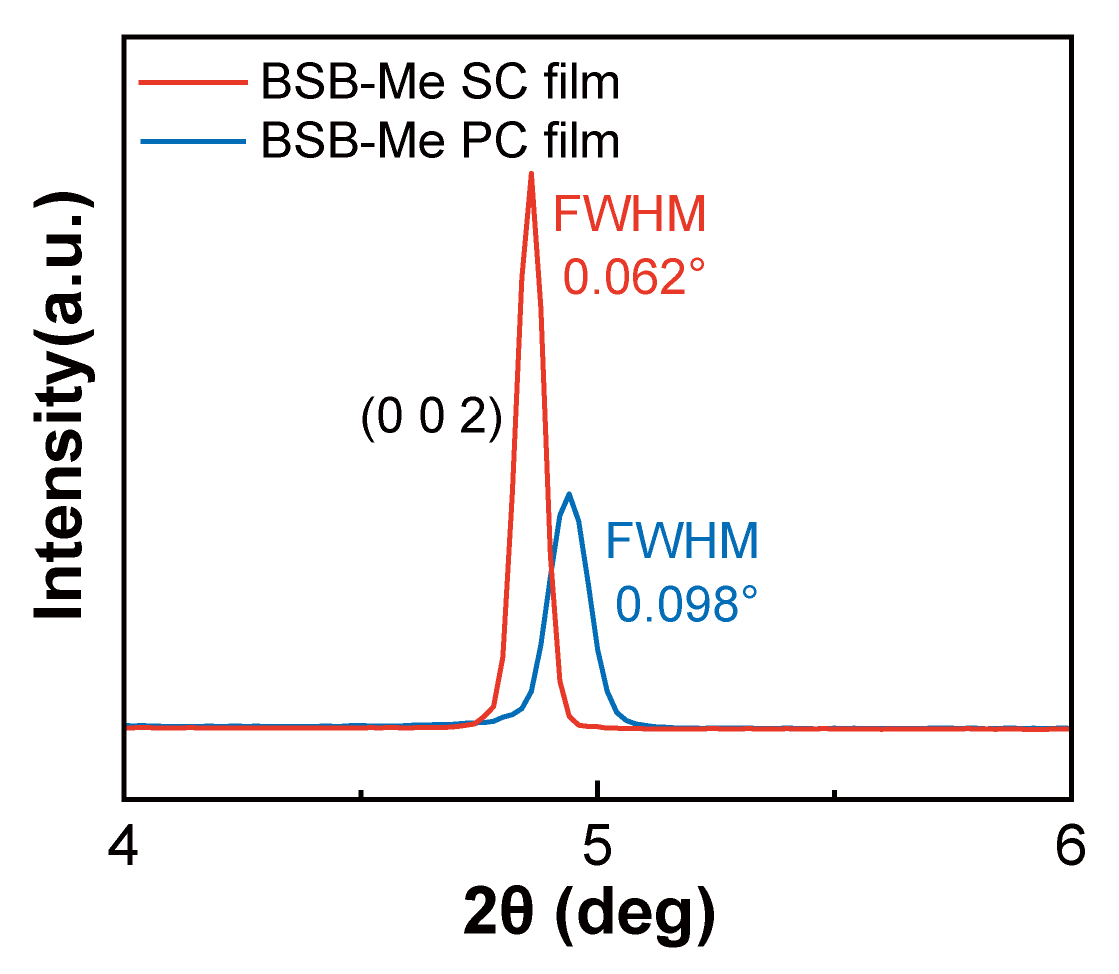
**

**Figure S1.** More-detailed XRD diffraction of BSB-Me SC and PC films corresponding to the (002) plane. The BSB-Me SC shows an intense and sharp narrow (002) peak at 2θ = 4.86 º (red), whereas BSB-Me PC film shows less intense and broad peak at 2θ = 4.94 º (blue). On the basis of different growing process, this upshift of the (002) peak can be ascribed to the lower crystallinity of BSB-Me PC films.

**
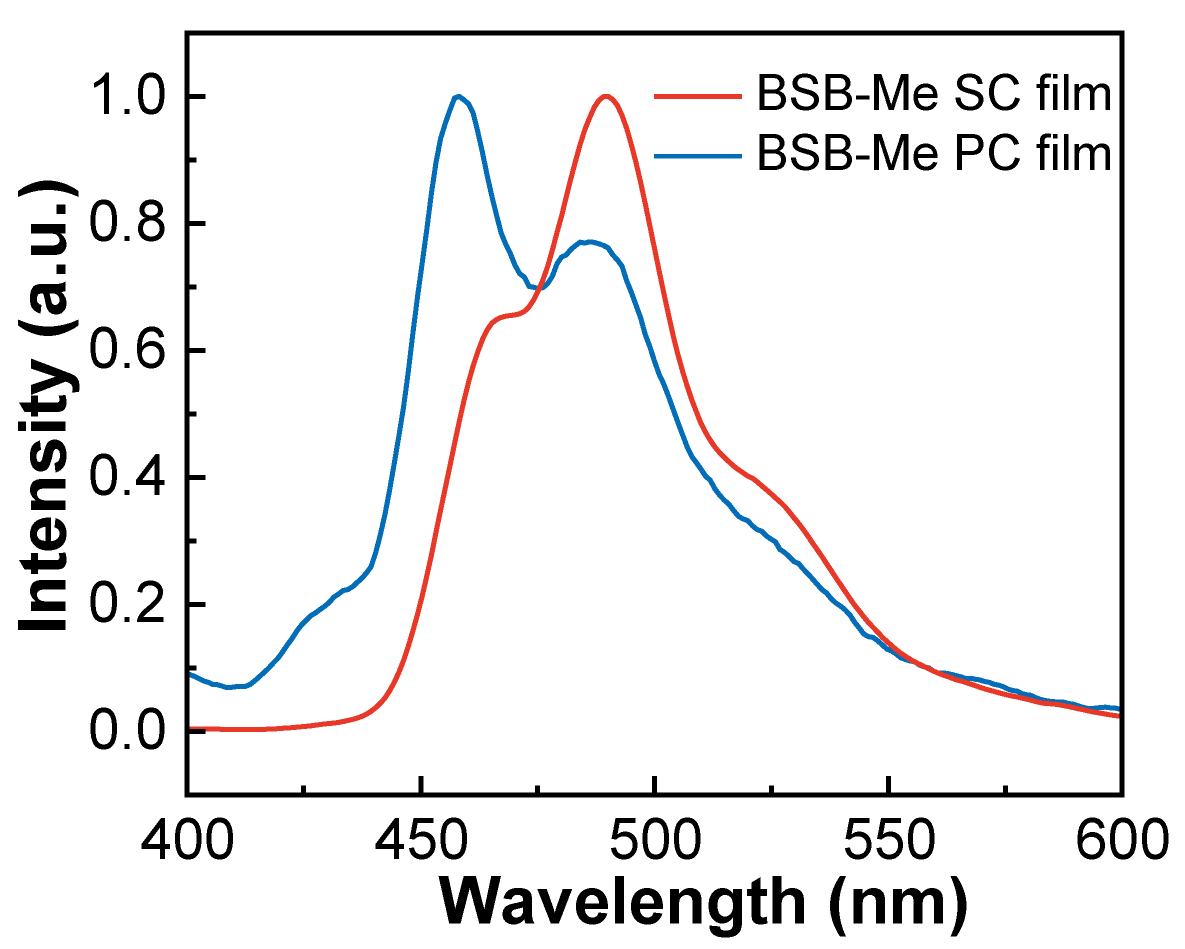
**

**Figure S2.** The PL spectra of BSB-Me SC and PC films show a thickness-dependent PL behavior, which can be ascribed to the re-absorption by ground-state molecules.^1^ The BSB-Me PC films with a thickness of only 40 nm showed a peak wavelength at 461 nm due to the smaller re-absorption effect. For the thick BSB-Me SC films (~400 nm), the peak wavelength occurred at 488 nm that was ascribed to the larger re-absorption effect.

**
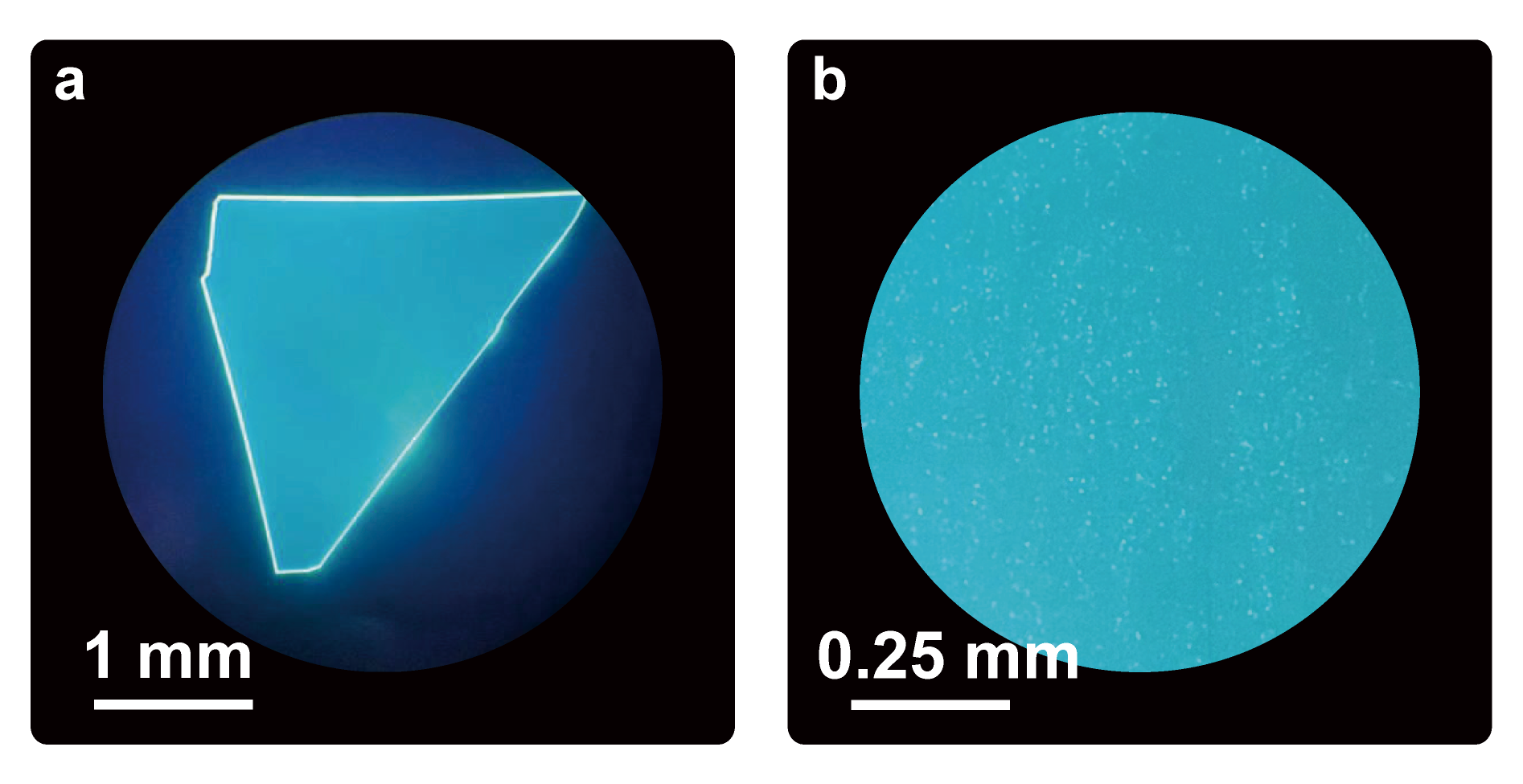
**

**Figure S3.** Representative fluorescence images of BSB-Me SC (**a**) and PC (**b**) films illuminated under a UV light irradiation.


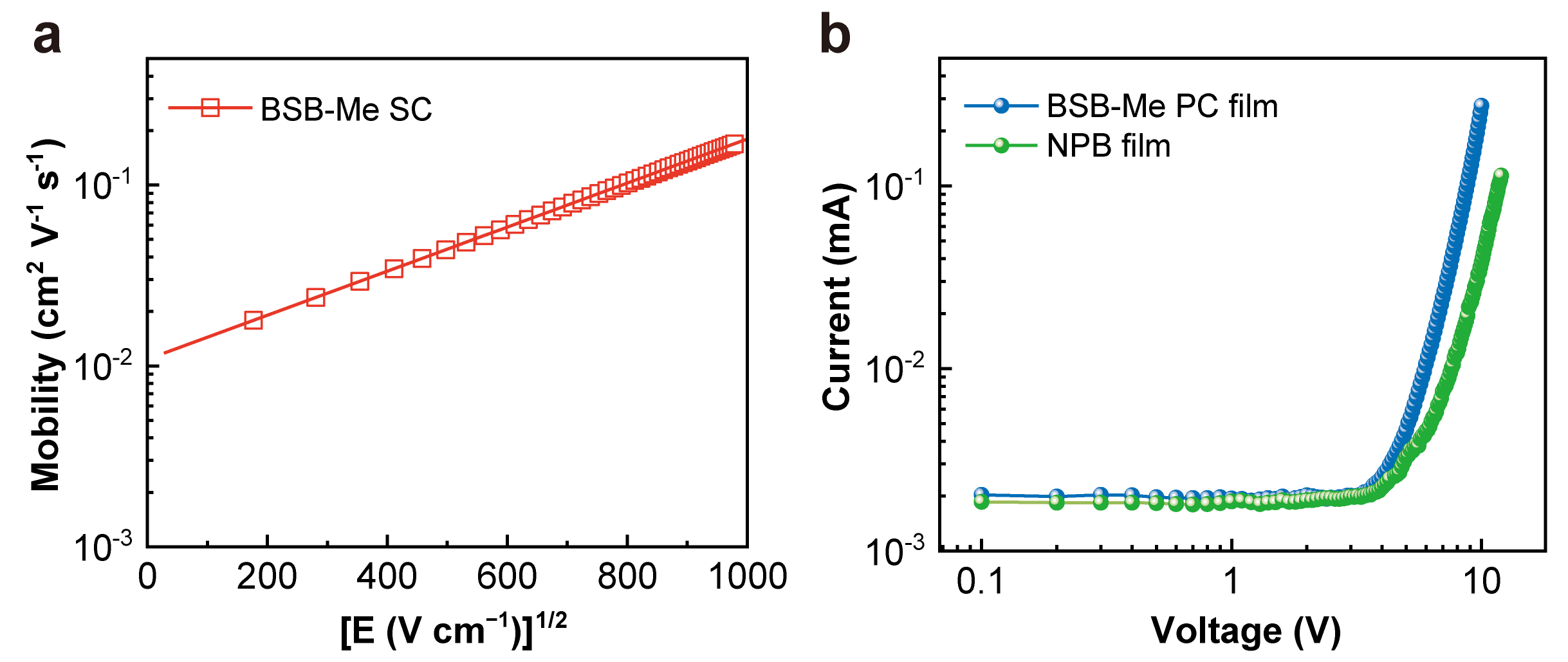


**Figure S4.** (**a**) Field dependent hole mobility of BSB-Me SC film estimated by SCLC method. (**b**) The current-voltage curves of BSB-Me PC and amorphous NPB films by SCLC method based on the hole-only devices.


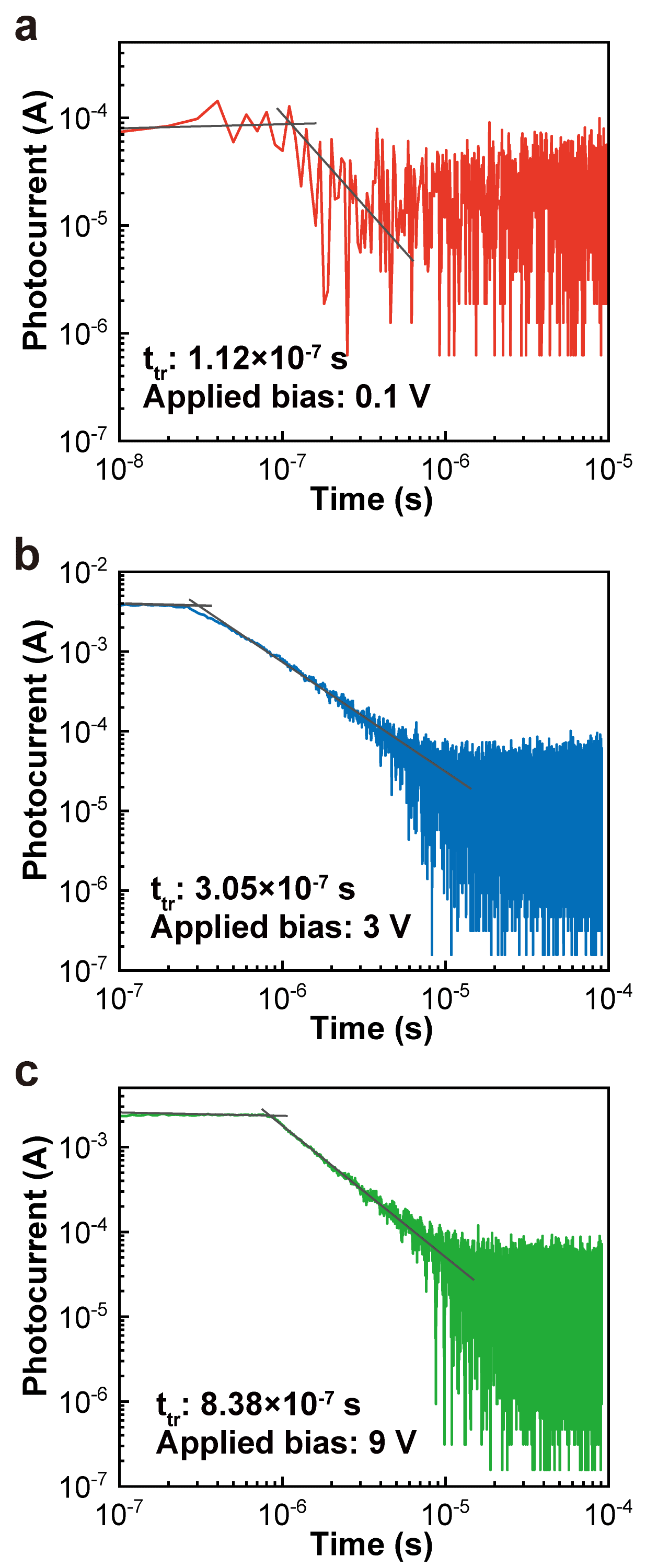


**Figure S5.** Time-of-flight transient photocurrent spectra of BSB-Me SC (**a**), BSB-Me PC (**b**), and amorphous NPB (**c**) films.


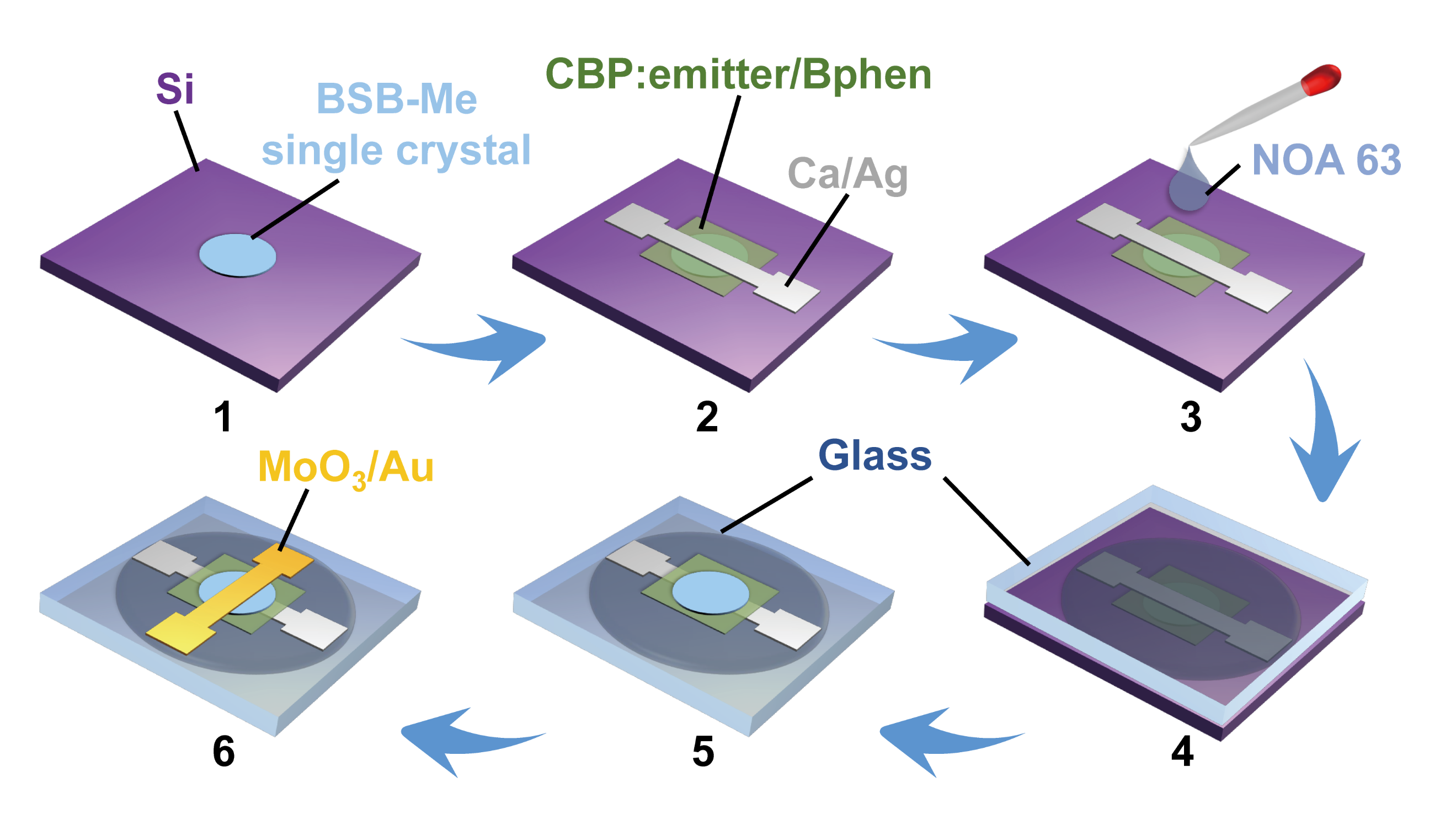


**Figure S6.** The schematic diagram of fabrication for SC-OLEDs by the template stripping method.


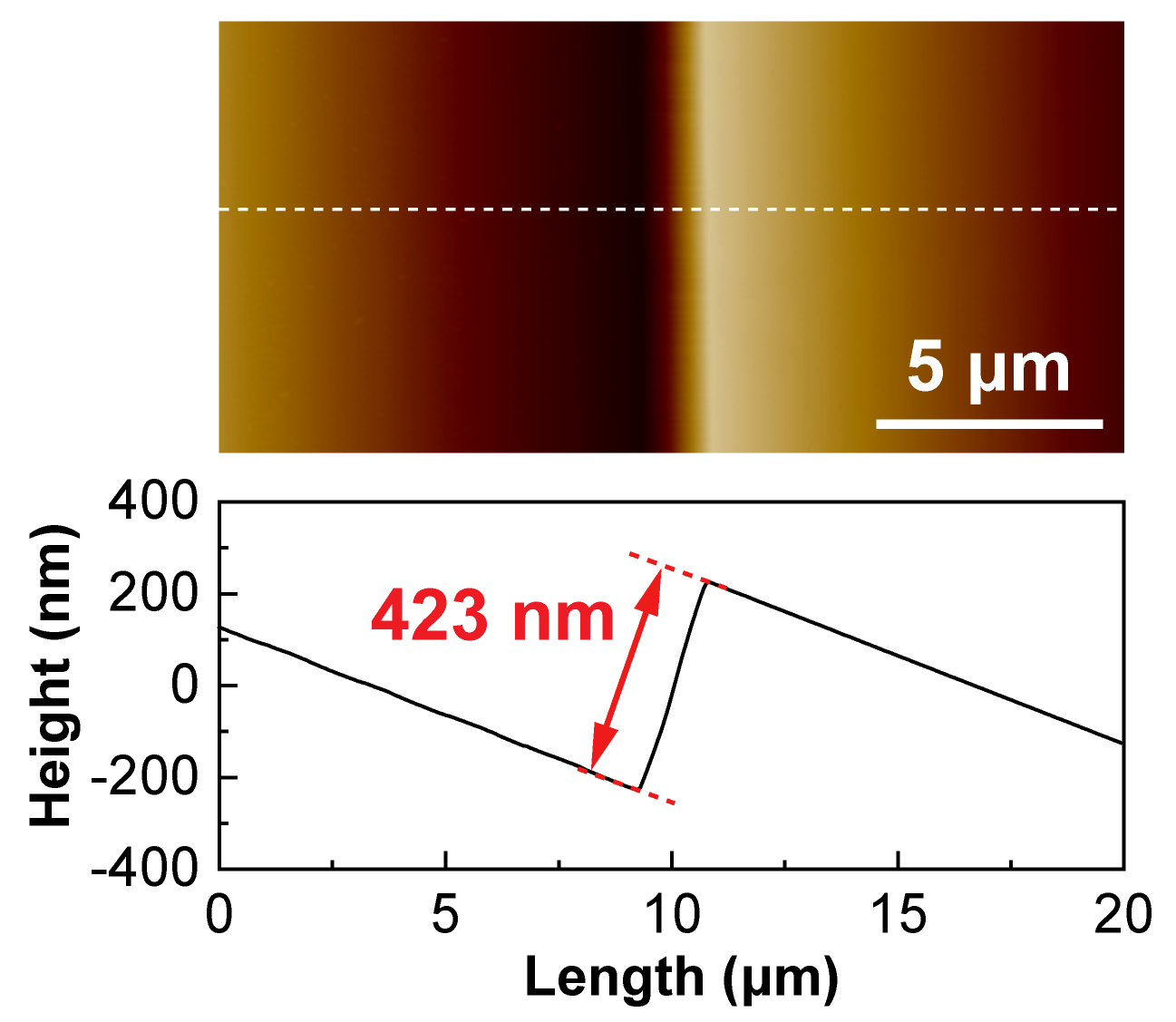


**Figure S7.** AFM image of the BSB-Me SC film with height profile.


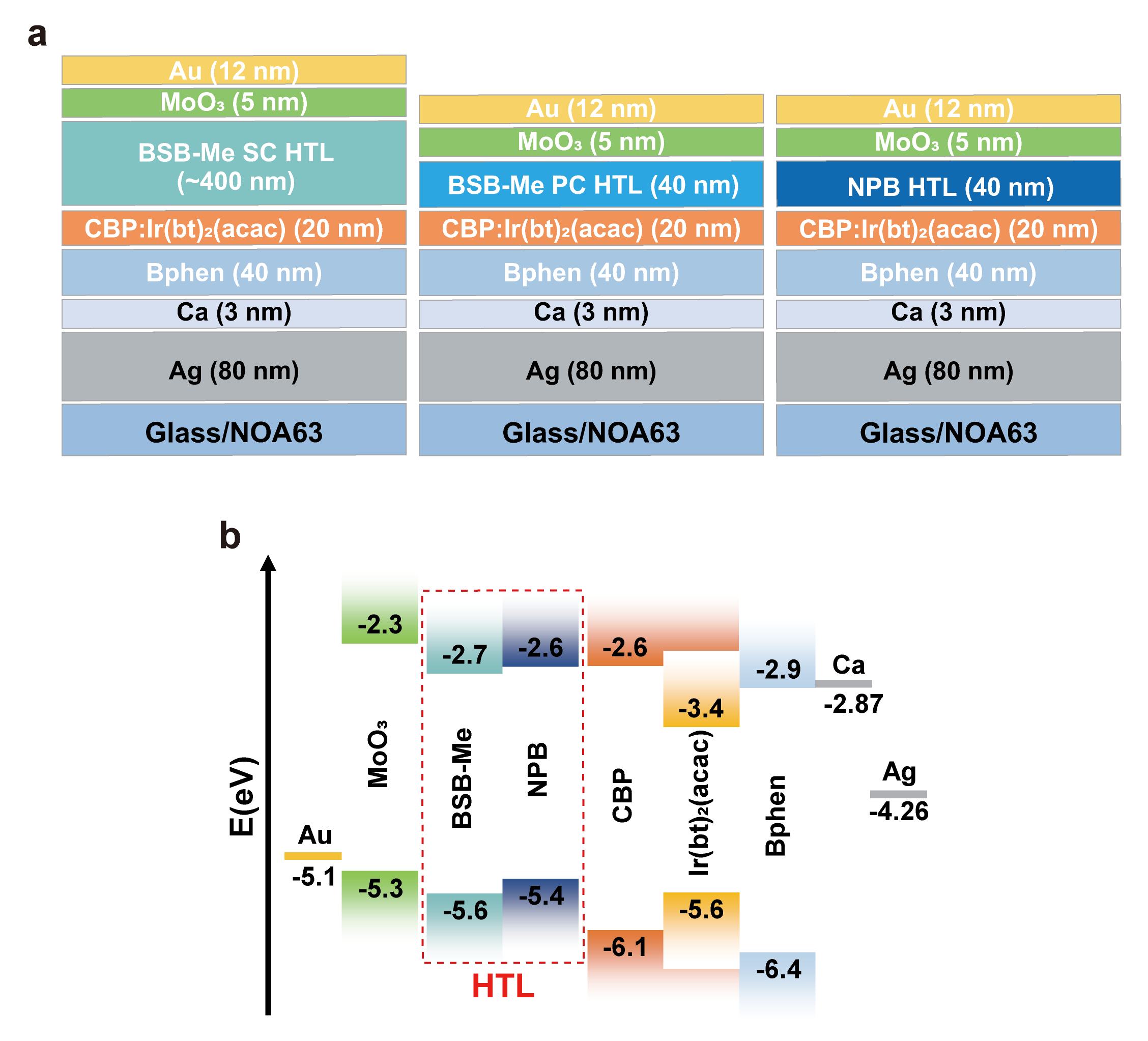


**Figure S8.** (**a**) Schematic illustration of the OLED structures with different HTLs. (**b**) Energy-level diagram of the OLEDs with different HTLs.


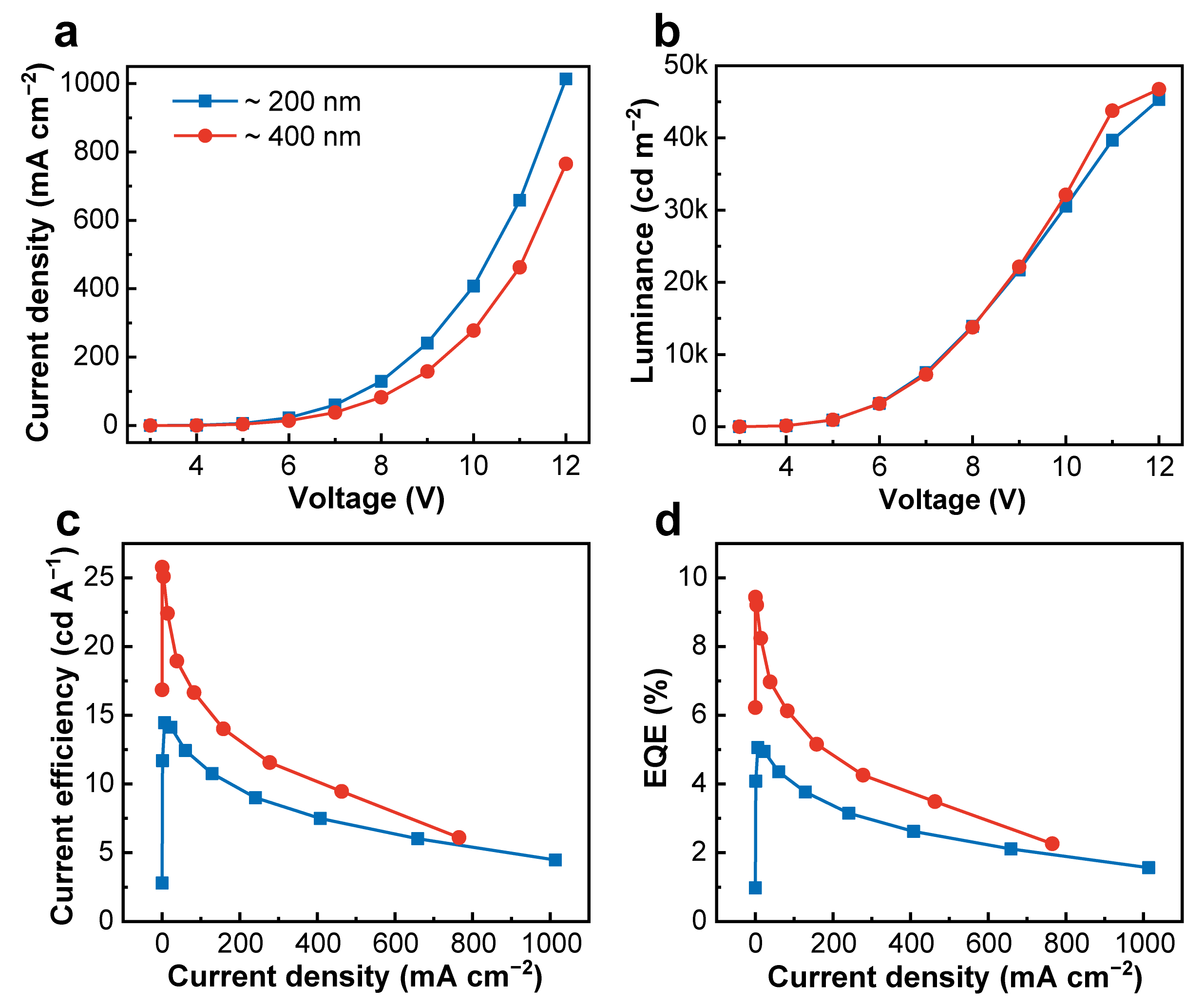


**Figure S9.** EL performances of SC-OLEDs with different BSB-Me SC-HTL thicknesses. Plots of current density-voltage (**a**), luminance-voltage (**b**), current efficiency-current density (**c**), and EQE-current density (**d**) of SC-OLEDs with different SC-HTL thicknesses.


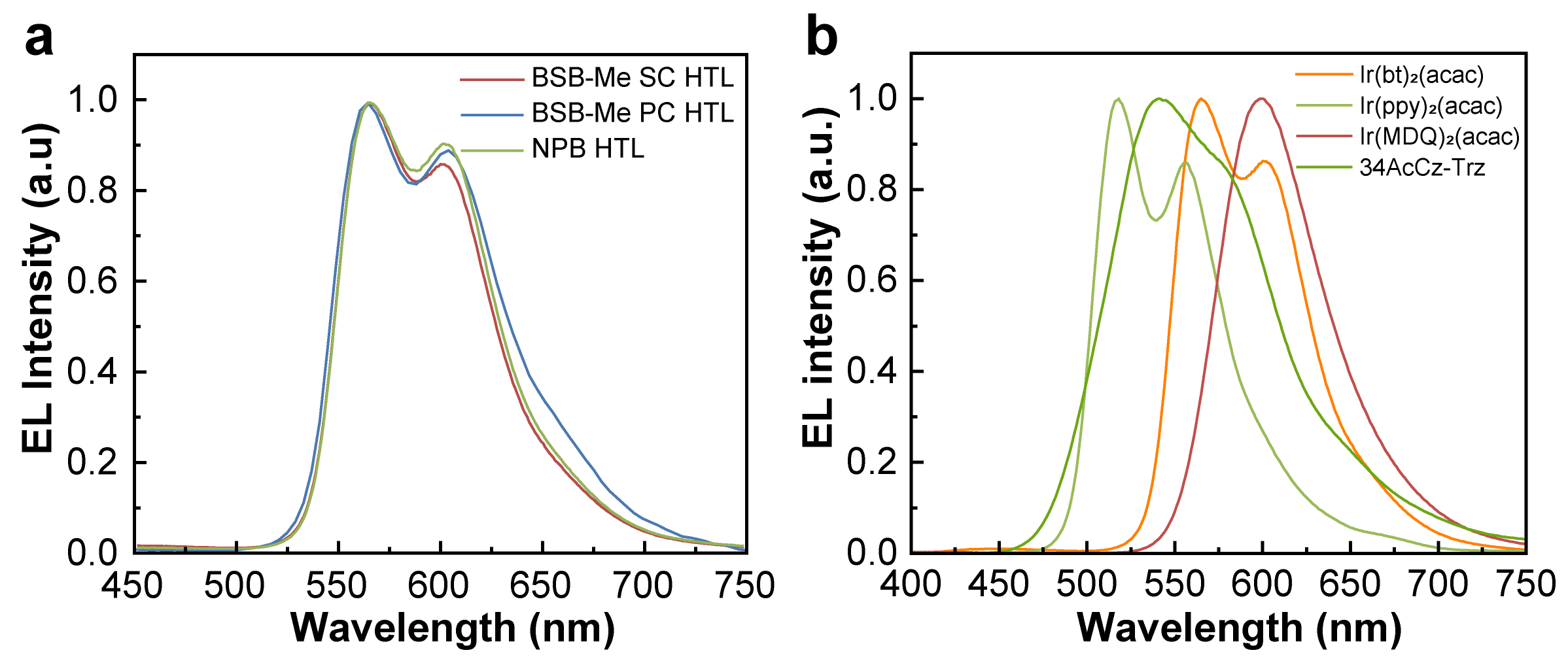


**Figure S10.** The corresponding EL spectra of OLEDs based on different HTLs with Ir(bt)_2_(acac) emitter (**a**), and different emitters with BSB-Me SC HTL (**b**).

**
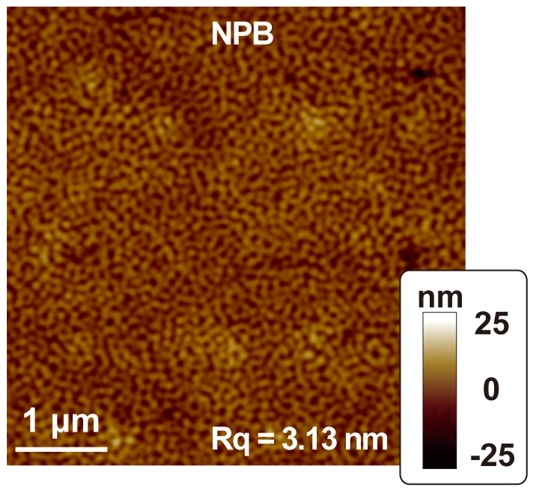
**

**Figure S11.** AFM images of original amorphous NPB film with an RMS roughness value of 3.13 nm.


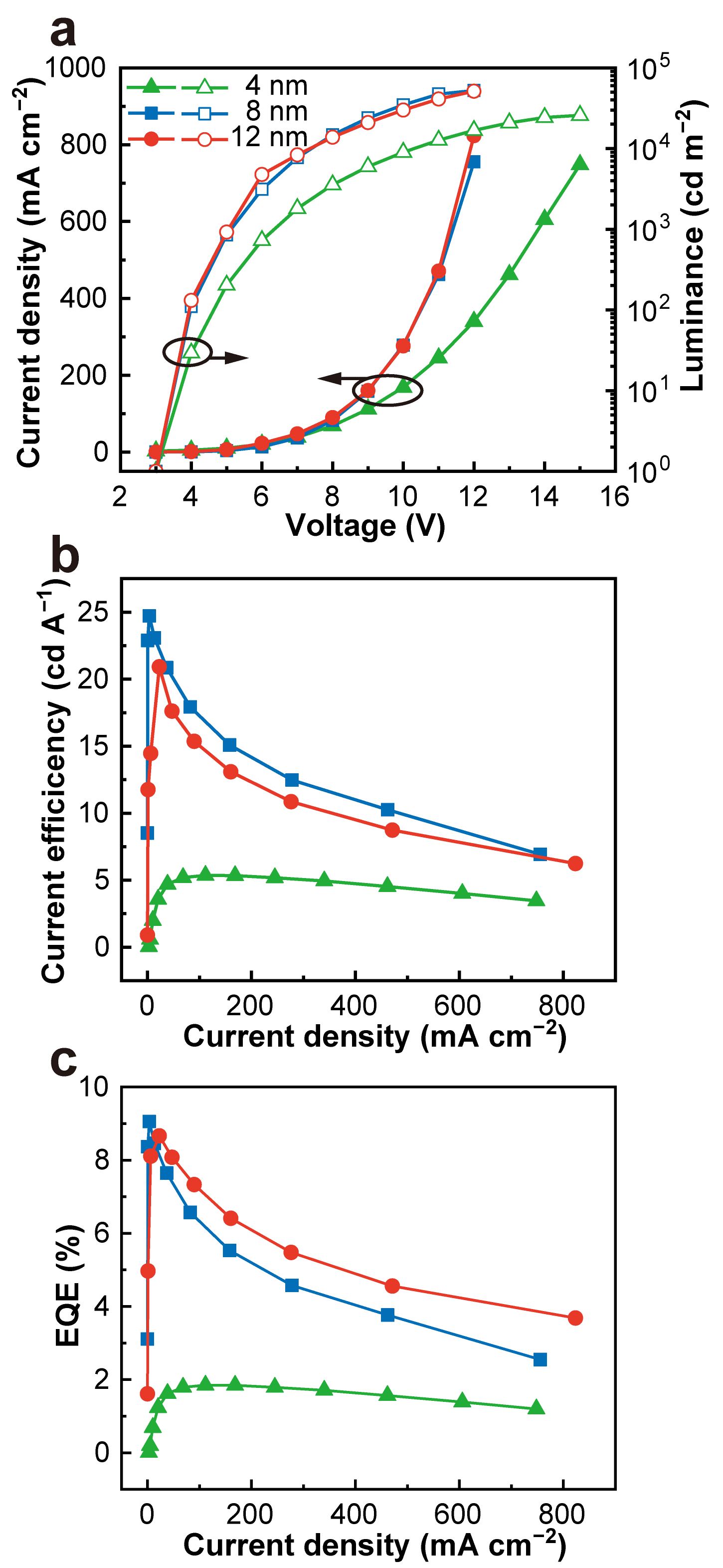


**Figure S12.** EL performances of BSB-Me SC HTL-based OLEDs with 12-, 8-, and 4-nm-thick Au anodes. (**a**) Current density-voltage-luminance, (**b**) current efficiency-current density, (**c**) EQE-current density curves of BSB-Me SC HTL-based OLEDs with 12-, 8-, and 4-nm-thick Au anodes.

**
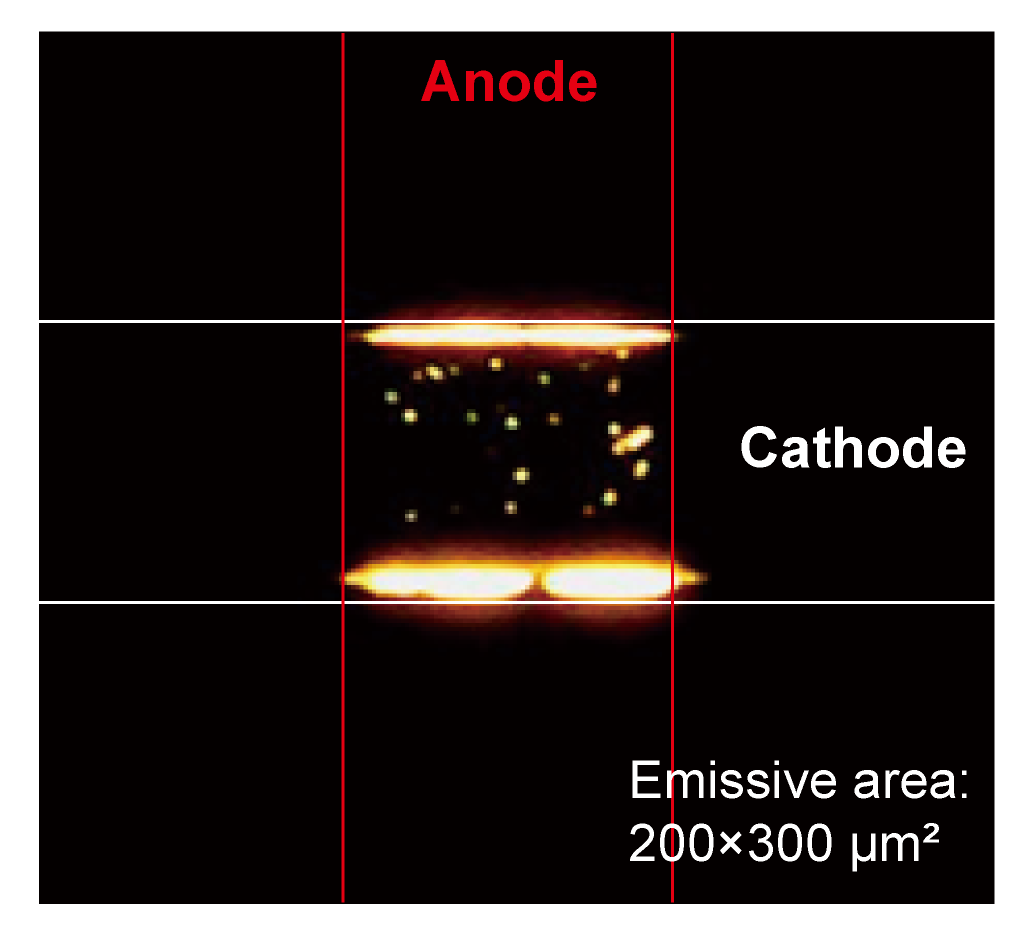
**

**Figure S13.** The photograph of the failed NPB HTL-based OLEDs with 8-nm-thick Au anode.

**
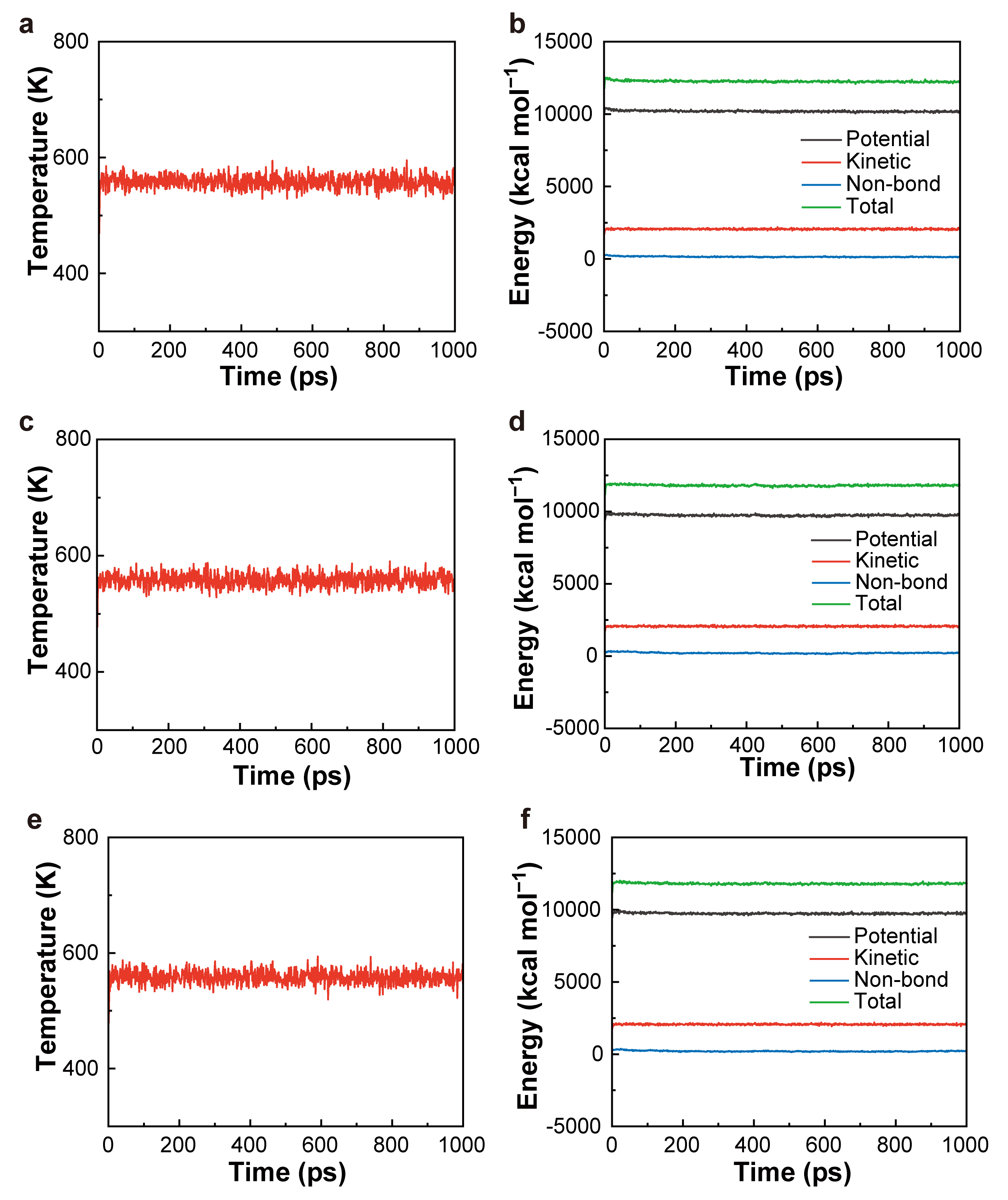
**

**Figure S14.** Temperature and energy fluctuation curves of the established model of interaction system of BSB-Me SC and CBP layer (**a**, **b**), BSB-Me PC and CBP layer (**c**, **d**), and amorphous NPB and CBP layer (**e**, **f**).


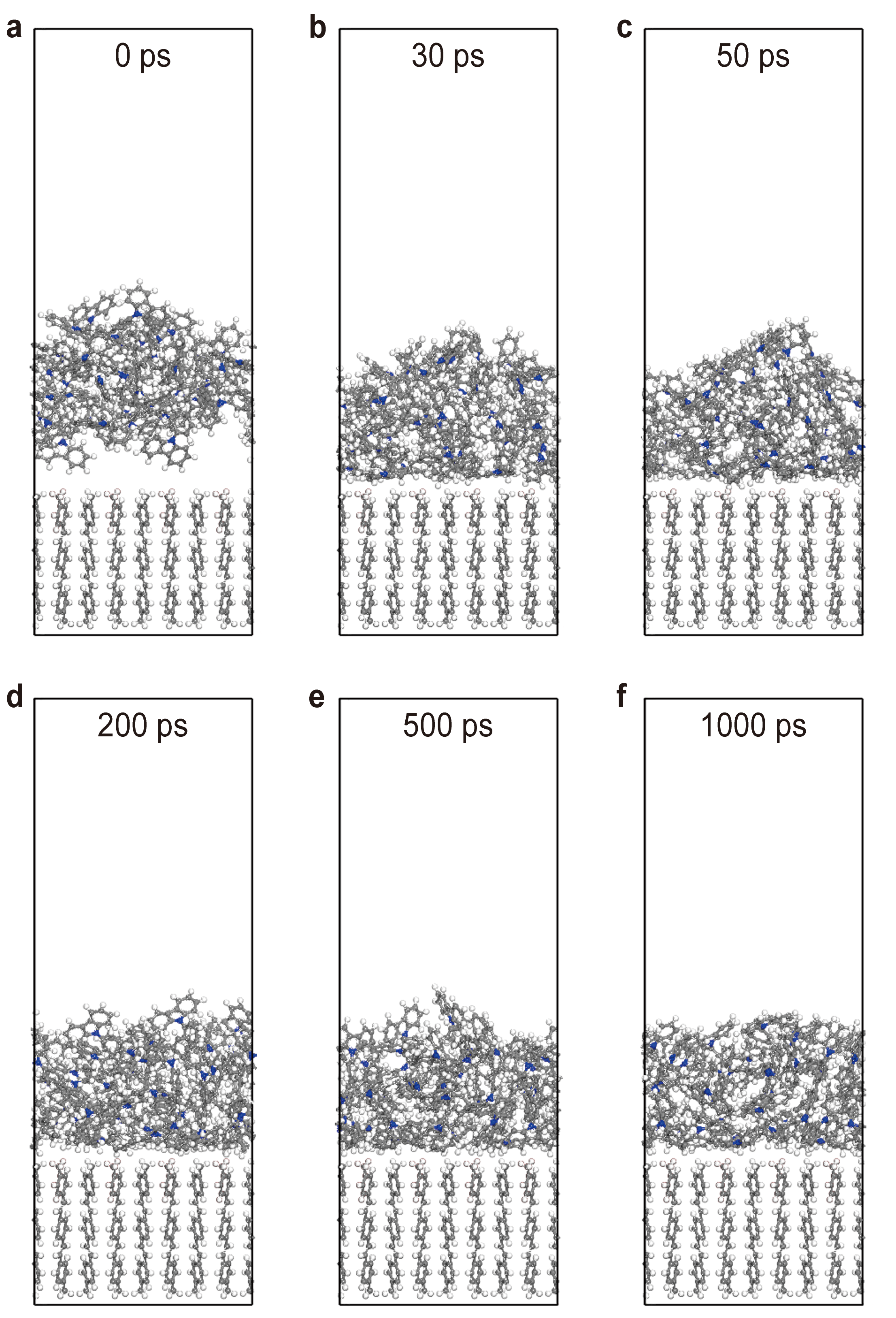


**Figure S15.** The atomic configurations of the BSB-Me SC/CBP interaction system at different moments (**a-f**: 0, 30, 50, 200, 500, and 1000 ps).


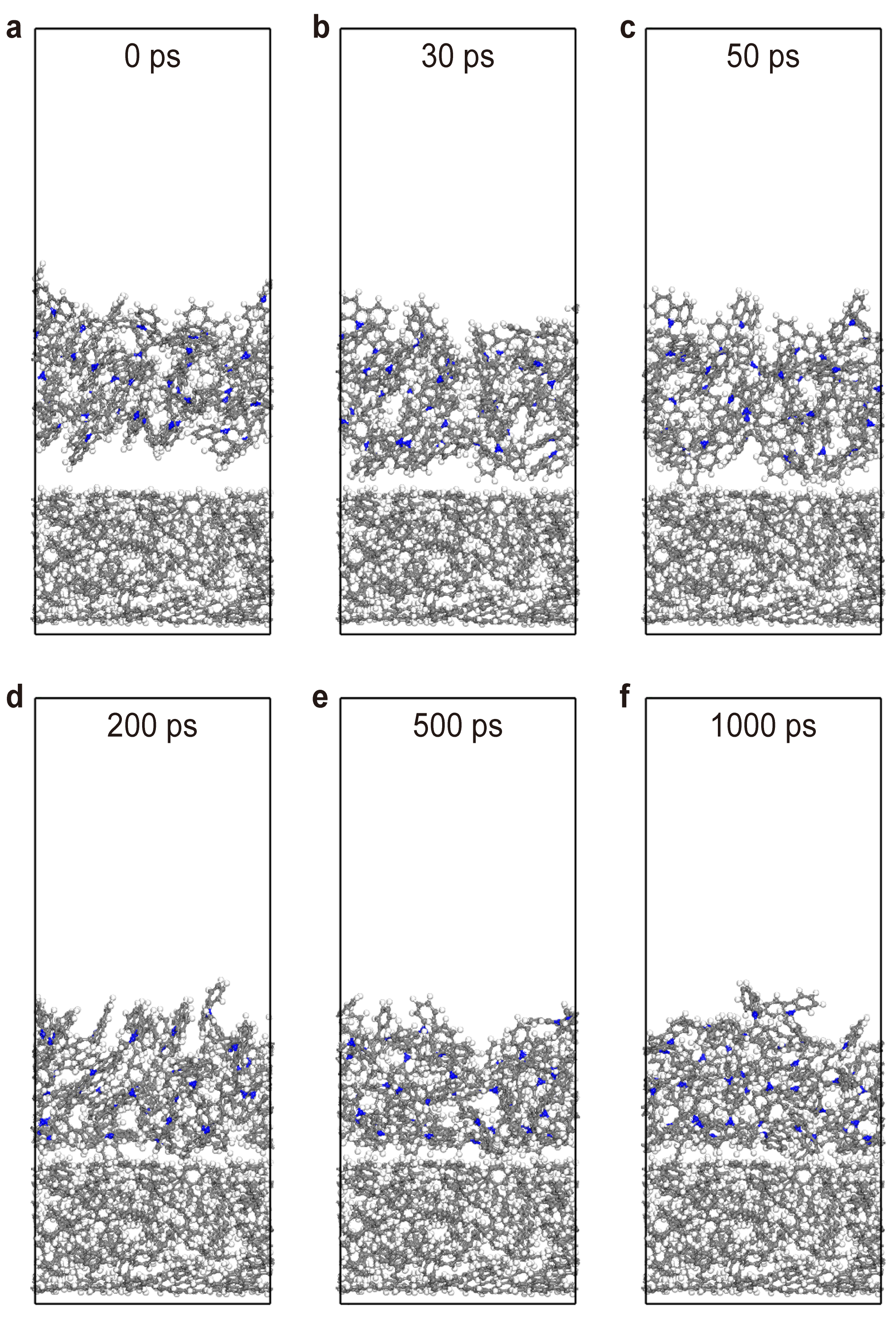


**Figure S16.** The atomic configurations of the BSB-Me PC/CBP interaction system at different moments (**a-f**: 0, 30, 50, 200, 500, and 1000 ps).


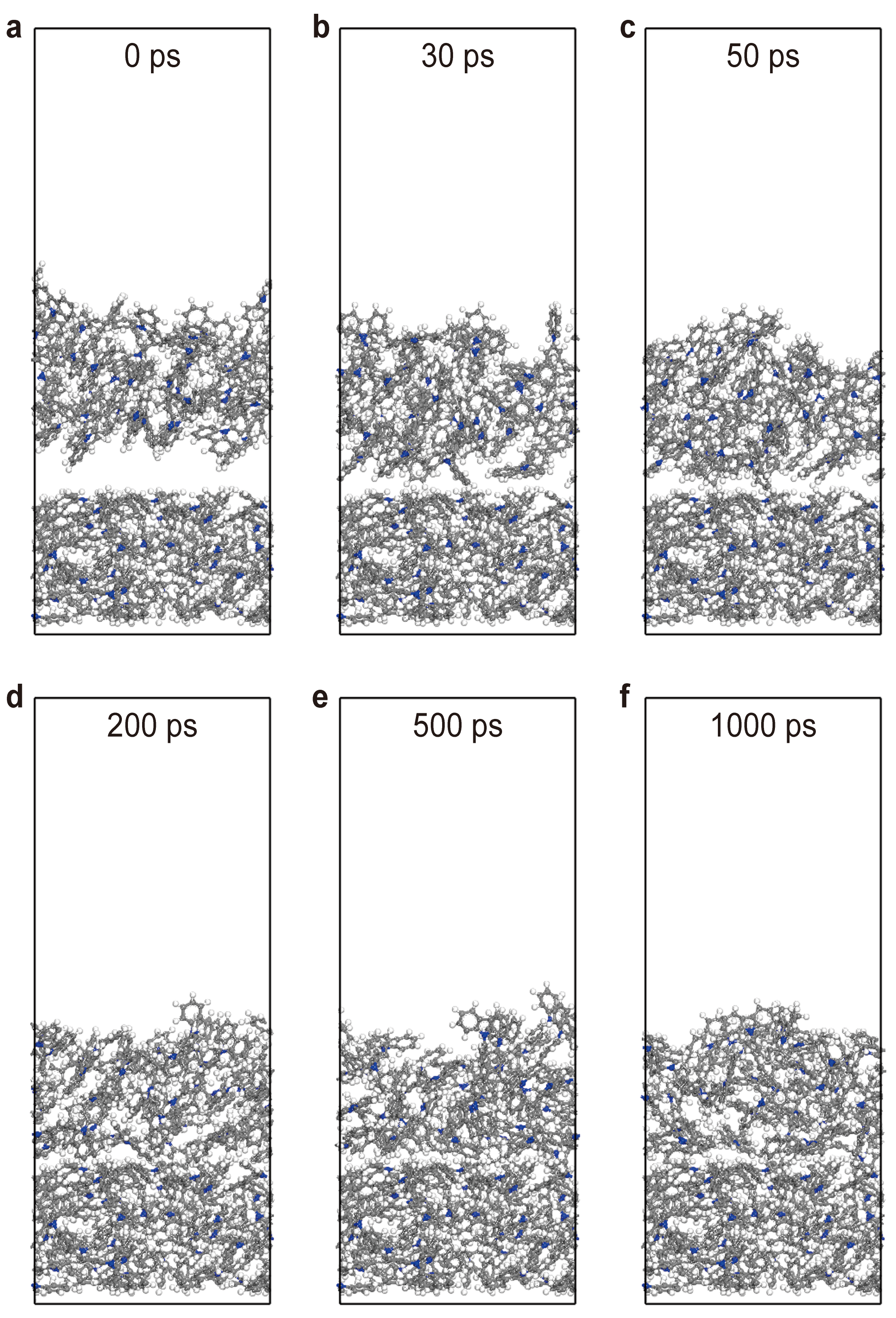


**Figure S17.** The atomic configurations of the amorphous NPB/CBP interaction system at different moments (**a-f**: 0, 30, 50, 200, 500, and 1000 ps).


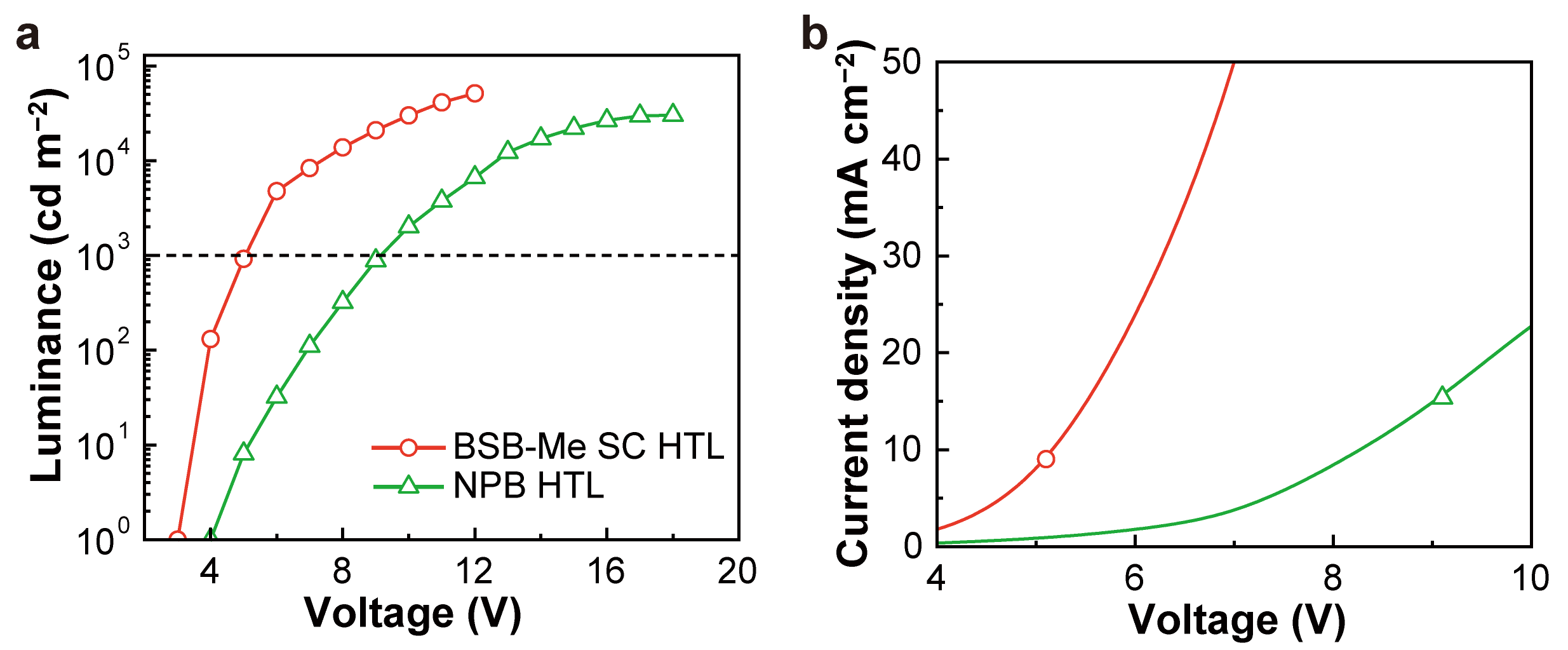


**Figure S18.** Luminance-voltage and current density-voltage curves of the OLEDs based on BSB-Me SC (**a**) and amorphous NPB (**b**) HTLs.


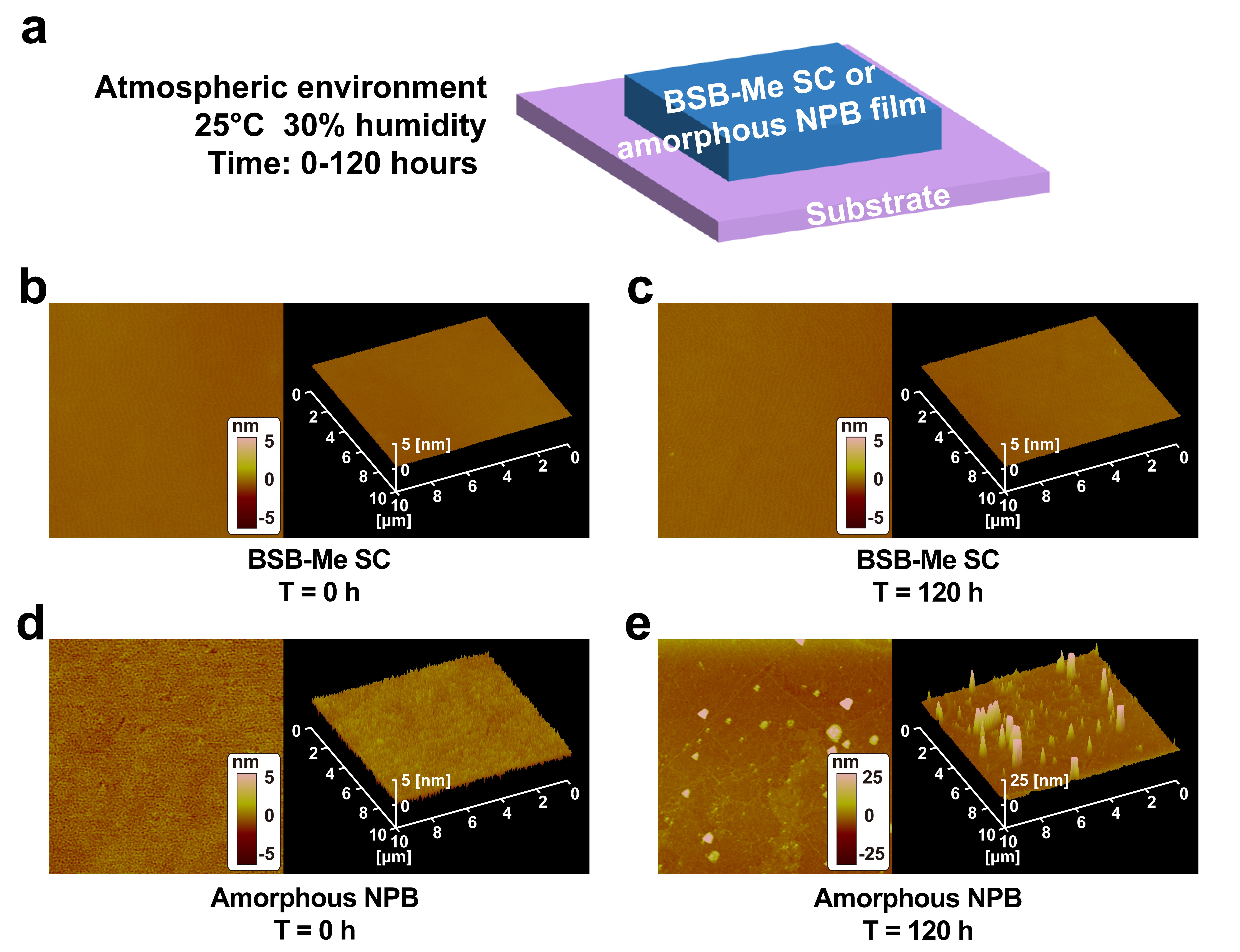


**Figure S19.** Morphological stability of both BSB-Me SC and amorphous NPB films at the atmospheric environment (25 °C and 30% humidity). AFM images of BSB-Me SC and amorphous NPB films at different time of T = 0 h (**b**, **d**), T = 120 h (**c**, **e**).


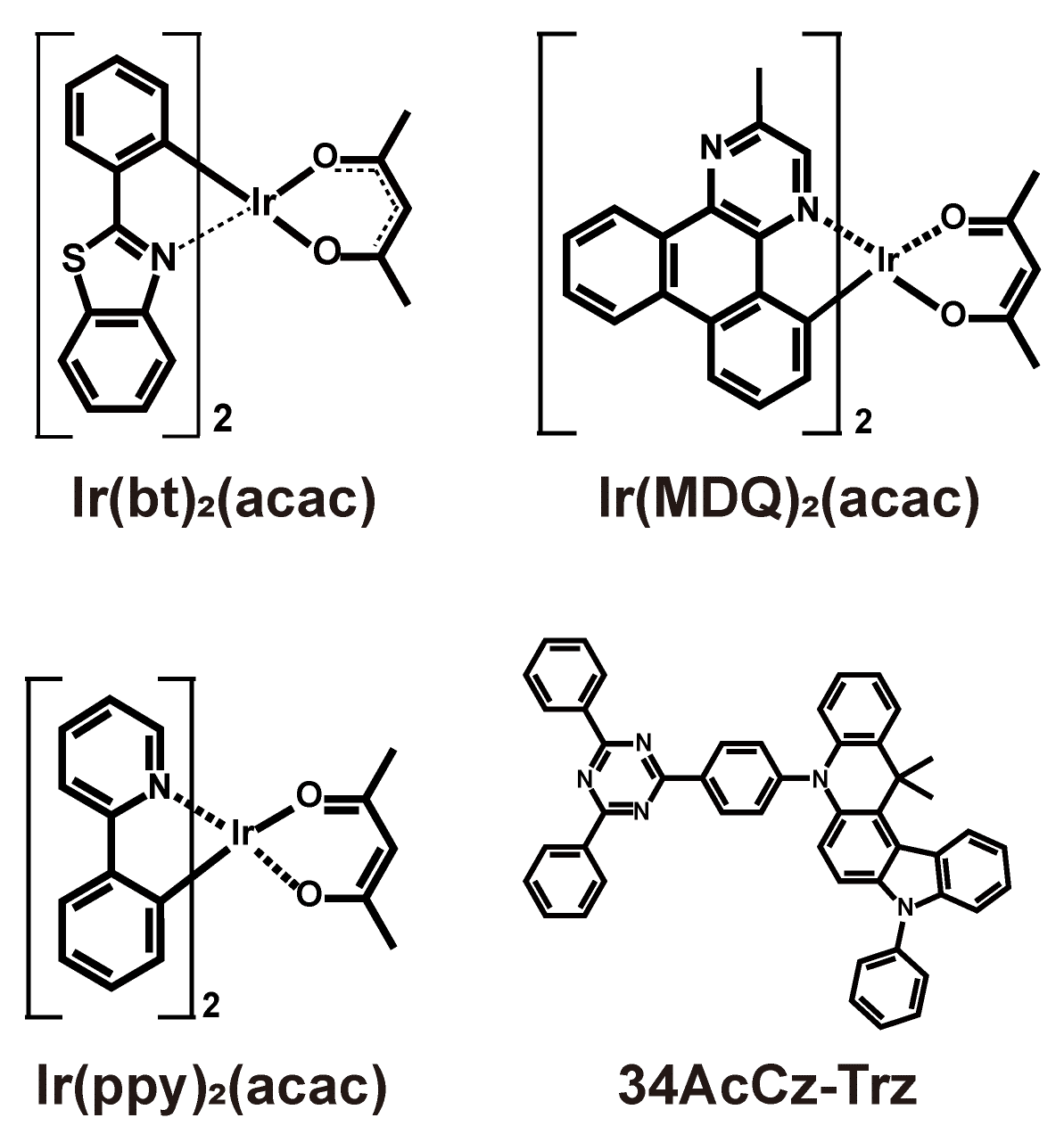


**Figure S20.** The molecular formula of the phosphorescence- and TADF-based emitters used in the SC-OLEDs.


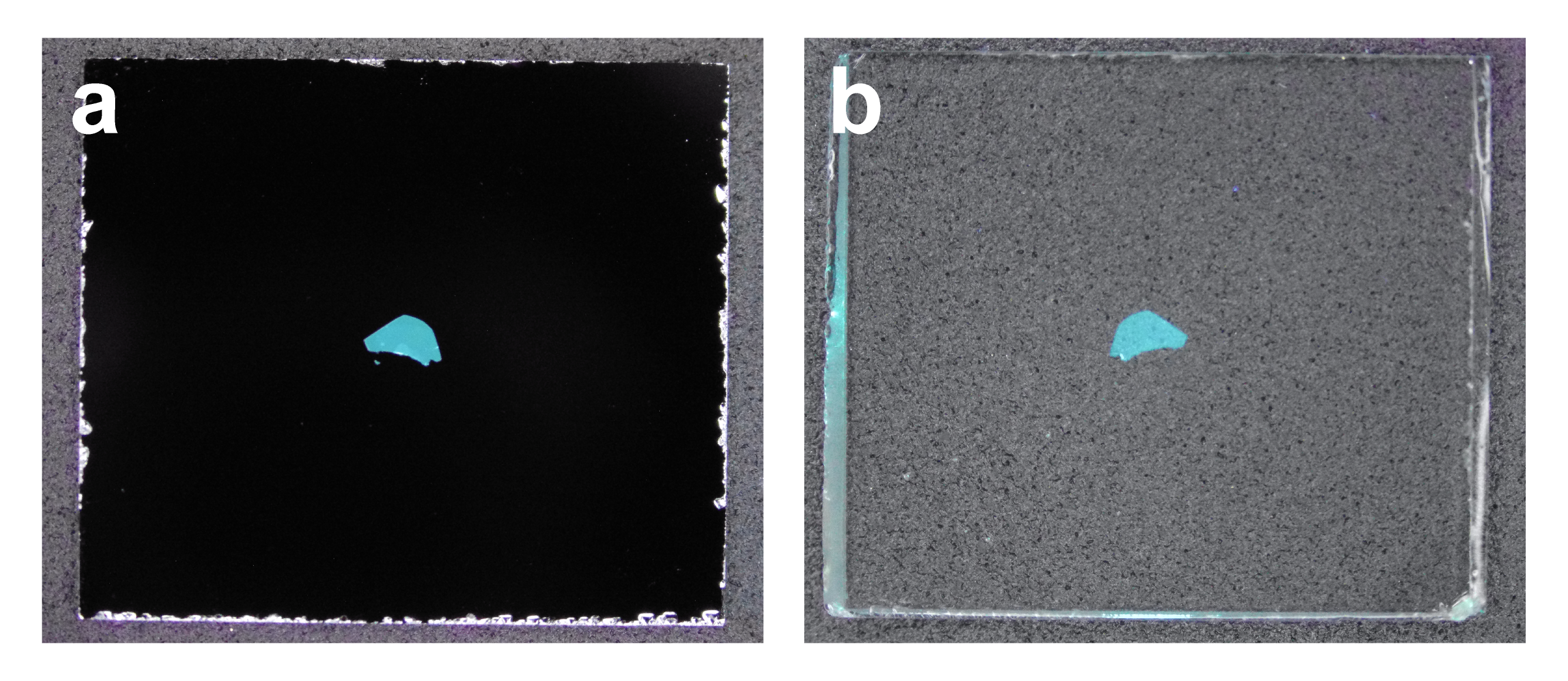


**Figure S21.** Optical images of BSB-Me SC film on the SiO2/Si substrate (**a**), and then stripped and transferred to the glass substrate via NOA63 photoresist (**b**).

**Supplementary Tables**

**Table S1.** Summary of the film thickness, transit time, applied bias, and hole mobilities of BSB-Me SC, BSB-Me PC, and amorphous NPB films.

| **Sample** | **Thickness**  **(nm)** | **Applied bias**  **(V)** | **Transit time**  **(s)** | **Hole mobility**  **(cm^2^ V^−1^ s^−1^)** |
| --- | --- | --- | --- | --- |
| **BSB-Me SC** | 490 | 0.1 | 1.12×10^−7^ | 0.21 |
| **BSB-Me PC** | 500 | 3 | 3.05×10^−7^ | 2.73×10^−3^ |
| **NPB** | 500 | 9 | 8.38×10^−7^ | 3.31×10^−4^ |

**Table S2.** The key performances of previous researches on OLEDs and organic light-emitting field-effect transistors (OLETs) based on organic single-crystalline (SC) and polycrystalline (PC) films.

| **Material** | **Material type** | **Device configuration** | **Luminance**  **(cd m**^−^**^2^)** | **CE**  **(cd A**^−1^**)** | **EQE**  **(%)** | **Ref.** |
| --- | --- | --- | --- | --- | --- | --- |
| dNaAnt | SC | OLET | 3180 | [-] | 1.75 | [4] |
| NBTA | SC | OLET | [-] | [-] | 2.02 | [5] |
| PBNA | SC | OLET | [-] | [-] | 3.63 | [6] |
| BSB-Me: Te: Pe | SC | OLED | 793 | 0.89 | 0.48 | [7] |
| BSB-Me: BTPB: Pe | SC | OLED | 5467 | 2.82 | 1.64 | [8] |
| BP1T-CN | SC | OLED | 6122 | 1.86 | 1.44 | [9] |
| *p*-6P | PC | OLED | ~20000 | 7.49 | 2.41 | [10] |
| BP1T | PC | OLED | 13900 | 2.42 | 4.70 | [11] |
| BSB-Me: Ir(bt)_2_(acac) | SC | OLED | 52270 | 24.73 | 9.06 | This work |
| BSB-Me: Ir(ppy)_2_(acac) | SC | OLED | 61930 | 38.03 | 10.50 | This work |
| BSB-Me: Ir(MDQ)_2_(acac) | SC | OLED | 20080 | 20.79 | 12.64 | This work |
| BSB-Me: 34AcCz-Trz | SC | OLED | 58280 | 37.75 | 11.80 | This work |

**Table S3.** Summary of the Haacke’s FoM as a function of transmittance wavelength corresponding to EL emission peaks of Ir(ppy)_2_(acac), 34AcCz-Trz, Ir(bt)_2_(acac), and Ir(MDQ)_2_(acac) emitters.

| **Emitter** | **Wavelength**  **(nm)** | **FoM (Ω^−1^)** | | | |
| --- | --- | --- | --- | --- | --- |
|  |  | BSB-Me SC | | Amorphous NPB | |
|  |  | 8-nm-Au | 12-nm-Au | 8-nm-Au | 12-nm-Au |
| Ir(ppy)_2_(acac) | 518 | 3.64 × 10^−3^ | 3.52 × 10^−3^ | 8.29 × 10^−4^ | 9.76 × 10^−4^ |
| 34AcCz-Trz | 540 | 4.12 × 10^−3^ | 4.03 × 10^−3^ | 1.07 × 10^−3^ | 1.12 × 10^−3^ |
| Ir(bt)_2_(acac) | 564 | 4.66 × 10^−3^ | 3.52 × 10^−3^ | 1.07 × 10^−3^ | 1.12 × 10^−3^ |
| Ir(MDQ)_2_(acac) | 600 | 4.66 × 10^−3^ | 2.68 × 10^−3^ | 1.21 × 10^−3^ | 9.76 × 10^−4^ |

**Table S4.** Summary of the energy loss from Joule-heat of series resistance of the OLEDs based on BSB-Me SC and amorphous NPB HTLs at about 1000 cd m^−2^.

| **Device** | **Voltage**  **(V)** | **Current density**  **(mA cm^−2^)** | **Input power**  **(mW cm^−2^)** | **Series resistance**  **(kΩ cm^−2^)** | **Series-resistance Joule-heat loss**  **(mW cm^−2^)** | **Ratio of series-resistance Joule-heat loss to input power (%)** |
| --- | --- | --- | --- | --- | --- | --- |
| **BSB-Me SC HTL** | 5.1 | 8.03 | 40.95 | 0.0835 | 5.38 | 13.14 |
| **NPB HTL** | 9.1 | 15.36 | 139.78 | 0.1344 | 31.71 | 22.67 |

**References**

1. Kabe, R. et al. Effect of Molecular Morphology on Amplified Spontaneous Emission of Bis-Styrylbenzene Derivatives. *Advanced Materials* **21**, 4034-4038 (2009).
2. Chu, T. Y., Song, O. K. Hole mobility of *N,N′*-bis(naphthalen-1-yl)-*N,N′*-bis(phenyl) benzidine investigated by using space-charge-limited currents. *Applied Physics Letters* **90**, 203512 (2007).
3. Matsushima, T. et al. High performance from extraordinarily thick organic light-emitting diodes. *Nature* **572**, 502-506 (2019).
4. Qin, Z. S. et al. High-Efficiency Single-Component Organic Light-Emitting Transistors. *Advanced Materials* **31**, 1903175 (2019).
5. Wan, Y. J. et al. Efficient Organic Light-Emitting Transistors Based on High-Quality Ambipolar Single Crystals. *ACS Applied Materials & Interfaces* **12**, 43976-43983 (2020).
6. Liu, L. Q. et al. Lamellar Organic Light-Emitting Crystals Exhibiting Spectral Gain and 3.6% External Quantum Efficiency in Transistors. *ACS Materials Letters* **3**, 428-432 (2021).
7. Ding, R. et al. High-Color-Rendering and High-Efficiency White Organic Light-Emitting Devices Based on Double-Doped Organic Single Crystals. *Advanced Functional Materials* **29**, 1807606 (2019).
8. An, M. H. et al. Well-Balanced Ambipolar Organic Single Crystals toward Highly Efficient Light-Emitting Devices. *Advanced Functional Materials* **30**, 2002422 (2020).
9. An, M. H. et al. Highly polarized emission from organic single-crystal light-emitting devices with a polarization ratio of 176. *Optica* **9**, 121-129 (2022).
10. Yang, X. X., Feng, X., Xin, J. H., Wang, H. B. & Yan, D. H. High-performance crystalline organic light-emitting diodes based on multi-layer high-quality crystals. *Organic Electronics* **64**, 236-240 (2019).
11. Xin, J. H. et al. Doped crystalline thin-film deep-blue organic light-emitting diodes. *Journal of Materials Chemistry C* **9**, 2236-2242 (2021).
12. Yang, J. et al. An Efficient Blue-Emission Crystalline Thin-Film OLED Sensitized by “Hot Exciton” Fluorescent Dopant. *Advanced Science* **10**, 2203997 (2022).
